# Effect of water quality, sanitation, hand washing, and nutritional interventions on child development in rural Bangladesh (WASH Benefits Bangladesh): a cluster-randomised controlled trial

**DOI:** 10.1016/S2352-4642(18)30031-2

**Published:** 2018-04

**Authors:** Fahmida Tofail, Lia CH Fernald, Kishor K Das, Mahbubur Rahman, Tahmeed Ahmed, Kaniz K Jannat, Leanne Unicomb, Benjamin F Arnold, Sania Ashraf, Peter J Winch, Patricia Kariger, Christine P Stewart, John M Colford, Stephen P Luby

**Affiliations:** a International Centre for Diarrhoeal Disease Research, Bangladesh (icddr,b), Dhaka, Bangladesh; b School of Public Health, University of California Berkeley, Berkeley, CA USA; c Department of International Health, Johns Hopkins Bloomberg School of Public Health, Baltimore, MD, USA; d Department of Nutrition, University of California Davis, Davis, CA, USA; e Division of Infectious Diseases and Geographic Medicine, Stanford University Stanford, CA USA

## Abstract

**Background:**

Poor nutrition and hygiene make children vulnerable to delays in growth and development. We aimed to assess the effects of water quality, sanitation, handwashing, and nutritional interventions individually or in combination on the cognitive, motor, and language development of children in rural Bangladesh.

**Methods:**

In this cluster-randomised controlled trial, we enrolled pregnant women in their first or second trimester from rural villages of Gazipur, Kishoreganj, Mymensingh, and Tangail districts of central Bangladesh, with an average of eight women per cluster. Groups of eight geographically adjacent clusters were block-randomised, using a random number generator, into six intervention groups (all of which received weekly visits from a community health promoter for the first 6 months and every 2 weeks for the next 18 months) and a double-sized control group (no intervention or health promoter visit). The six intervention groups were: chlorinated drinking water; improved sanitation; handwashing with soap; combined water, sanitation, and handwashing; improved nutrition through counselling and provision of lipid-based nutrient supplements; and combined water, sanitation, handwashing, and nutrition. Here, we report on the prespecified secondary child development outcomes: gross motor milestone achievement assessed with the WHO module at age 1 year, and communication, gross motor, personal social, and combined scores measured by the Extended Ages and Stages Questionnaire (EASQ) at age 2 years. Masking of participants was not possible. Analyses were by intention to treat. This trial is registered with ClinicalTrials.gov, number NCT01590095.

**Findings:**

Between May 31, 2012, and July 7, 2013, 5551 pregnant women residing in 720 clusters were enrolled. Index children of 928 (17%) enrolled women were lost to follow-up in year 1 and an additional 201 (3%) in year 2. 4757 children were assessed at 1 year and 4403 at 2 years. At year 1, compared with the control group, the combined water, sanitation, handwashing, and nutrition group had a higher rate of attaining the standing alone milestone (hazard ratio 1·19, 95% CI 1·01–1 ·40), and the nutrition group had a higher rate of attaining the walking alone milestone (1·32, 95% CI 1·07–1·62). The combined water, sanitation, handwashing, and nutrition group had a higher rate of attaining the walking alone milestone than those in the water, sanitation, and handwashing group (1·29, 1·01–1·65). At 2 years, we noted beneficial effects in the combined EASQ score in all intervention groups, with effect sizes smallest in the water treatment group (difference 0·15, 95% CI 0·04 to 0·26 *vs* control) and largest in the combined water, sanitation, handwashing, and nutrition treatment group (0·37, 0·27–0·46).

**Interpretation:**

Improvements in water quality, handwashing, sanitation, or nutrition supported by intensive interpersonal communication, when delivered either individually or in combination, contributed to improvements in child development. A crucial next step is to establish whether similar effects can be achieved with reduced intensity of promoter contacts that could be supported in large-scale interventions.

**Funding:**

Bill & Melinda Gates Foundation.

## Introduction

Children growing up in poverty are exposed to multiple psychological, physiological, and environmental risk factors that shape their development. Poverty increases exposure to poor sanitation and hygiene, acute and chronic infection, poor nutrition, food insecurity, abuse and neglect, and stress.^1^, ^2^ These conditions can have strong and enduring effects on child development across many domains.^2^ Globally, millions of children experience delays in physical health and cognitive development because of their exposures to poverty and related issues, such as nutrition, health care, education, and lack of stimulating environment.^1^

Programmes and policies reducing exposures to risk factors or enhancing protective factors can improve the trajectories of children's development. The approaches with the strongest evidence base so far include nutrition counselling, provision of fortified food (eg, lipid-based nutritional supplementation), micronutrient supplem-entation, and parenting support and education.^3^ Although these options have been tested, with many positive results, there is little agreement on the optimal design of programmes to maximise growth and improve cognitive or language development for children in low-income countries. It is also unknown to what extent combinations of these interventions might be additive or synergistic.

**Research in context**

**Evidence before this study**

Evidence linking health and nutrition with child development was scarce before the start of the WASH Benefits trial. Therefore, we did not do a systematic review before starting our trial; our evidence base at that time was the reviews by Walker et al, Grantham-McGregor et al, and Engle et al in *The Lancet* Series on Child Development in 2007 and 2011. Later on we identified more updated systematic reviews that examined associations between childhood health status and cognitive development in low-income and middle-income countries, the most recent of which was published in 2016 (Black et al). We updated the search in PubMed to July 15, 2017, using the search terms “water”, “sanitation”, and “child development”, for publications in English, and found an additional cohort study (Dearden et al, 2017) including several countries showing that children with access to improved water and toilet facilities in their first year of life had higher language scores (receptive vocabulary) at age 5 and 8 years. Overall, the methodological rigor of studies linking water, sanitation, and handwashing interventions and child development was poor, and only one randomised controlled trial had been done (Bowen et al, 2012). In this study, children younger than 2 years living in squatter settlements in urban Karachi, Pakistan, were randomly assigned to receive soap and intensive handwashing promotion for 9 months. Their scores on the Battelle Developmental Inventory II, 5 years after intervention at 5–7 years of age were 0·4 SDs higher than control children who received no intervention and no visits. We also reviewed evidence linking nutrition interventions and child development, and found that direct micronutrient supplementation to deficient populations led to improved child development (Walker et al, 2011). There was no evidence before this study about lipid-based nutrient supplements and child development, but while this study was ongoing, new evidence emerged that these supplements might be beneficial to child development in Bangladesh, Burkina Faso, and Ghana, but not in Malawi.

**Added value of this study**

In this study, we assessed the single and combined effect of water, sanitation, handwashing, and nutrition interventions delivered with the support of frequent community worker visits on child development at age 1 and 2 years. We found some benefits in gross motor milestones when children were approximately 1 year old. We also found consistent developmental benefits in communication, gross motor, and personal social skills among children in all intervention groups when children were approximately 2 years old.

**Implications of all the available evidence**

Our findings suggest that interventions designed to improve water quality, sanitation, handwashing practices, or nutrition have cumulative beneficial effects on child development in addition to growth and reduced acute illness. Notably, we also recorded benefits of the single interventions in many cases, which would be a cheaper alternative to delivering combined interventions. Given that this trial was a test of efficacy, an important next step would be to consider how to bring these interventions to scale—both individually and combined—and to optimise effectiveness and cost-effectiveness.

Interventions targeting nutrition or development can be classified as development-specific interventions (ie, those addressing immediate determinants of nutrition and child development, such as inadequate nutrient intake and unsupportive caregiving practices), or development-sensitive interventions (ie, those addressing the underlying causes of undernutrition or poor development such as poverty, food insecurity, or scarcity of water and sanitation goods or services).^4^ Combined water, sanitation, and handwashing interventions fall into the broader category of nutrition-sensitive or development-sensitive interventions. The effects of these combined interventions on child development have not been extensively studied.

Exposure to poor water and sanitation causes diarrhoea in young children, and this is especially common in those living in low-income or middle-income countries.^5^ A recent meta-analysis examining effects of combined water, sanitation, and handwashing interventions on anthropometry showed small effects on improving linear growth in children younger than 5 years, and no effects on underweight or wasting.^6^ Findings of observational studies have shown associations between diarrhoea or other infectious diseases, and impaired cognitive outcomes.^7^, ^8^ One of the few randomised-controlled trials on this topic, done in Pakistan, found significant benefits of a handwashing promotion intervention on child development outcomes, but not on growth.^9^

The WASH Benefits trial in Bangladesh was designed together with a WASH Benefits trial in Kenya to assess the independent and combined effects of water, sanitation, handwashing, and nutrition interventions on child growth, health, and development after 2 years of intervention.^10^ In Bangladesh, children receiving sanitation, handwashing, nutrition, and all combined interventions had less caregiver-reported diarrhoea, and children receiving interventions with nutritional components had modestly improved growth when compared with children from control households.^11^ In the companion trial in Kenya, small improvements in growth were seen in children receiving interventions with nutritional components, but none of the interventions reduced diarrhoea prevalence;^12^ some improvement in motor development was seen after 1 year, but there was no beneficial effect in measured child development indicators across all intervention groups after 2 years.^10^

The objective of this analysis was to assess whether: interventions improving water quality; sanitation; hand-washing with soap; water, sanitation, and handwashing in combination; nutrition; or water, sanitation, and hand-washing, and nutrition in combination would improve indicators of child development during the first 2 years of life; and whether the combination of water, sanitation, handwashing, and nutrition would improve child development measurements more than combined water, sanitation, and handwashing or nutrition alone.

## Methods

### Study design

Details on the study methods and rationale have been published previously.^11^, ^13^ The Bangladesh WASH Benefits study was a cluster-randomised controlled trial done in the rural villages of Gazipur, Kishoreganj, Mymensingh, and Tangail districts of central Bangladesh. We specifically chose areas with low ground water iron and arsenic (because these variables can affect the chlorine-based intervention), and where no major water, sanitation, or focused nutrition programmes were underway or planned by the government or large non-government organisations. We defined a cluster as consisting of eight pregnant women who lived close enough to each other so that a community promoter could readily walk to each compound. We maintained a 1 km buffer around each cluster to minimise the potential for spillover between clusters. The clusters were randomly assigned to seven study groups: drinking water treatment and safe storage; sanitation; handwashing; combined water, sanitation, and handwashing; nutrition; combined water, sanitation, handwashing, and nutrition; and a double-sized control group, which received no intervention or health promoter visits.

The study protocol was approved by human subjects committees at icddr,b (PR-11063), the University of California, Berkeley (UC Berkeley), CA, USA (2011-09-3652), and Stanford University, CA, USA (25863).

### Participants

We enrolled pregnant women in their first or second trimester of pregnancy who intended to stay in their villages for 24 months post enrolment; their in utero children were considered to be the index children. After obtaining verbal and written informed consent, trained research assistants enrolled pregnant women in the study.

### Randomisation and masking

Eight adjacent clusters formed a geographical block. An offsite investigator from UC Berkeley (BFA) used a random number generator to block randomise clusters into one of six intervention groups or into the double-sized control group, providing geographically pair-matched randomisation. We used a larger control group to improve the precision in comparing each of the six intervention groups against control. Participants and other community members were informed of their intervention group assignment after the baseline survey and randomisation. Interventions included distinct visible components and community health promoter visits, which meant that neither the study participants nor the data collectors could be masked to intervention assignment. Data collectors were different from the intervention teams.

### Procedures

Community health promoters who had completed 8 or more years of formal education, lived within walking distance of an intervention cluster, and who had social acceptance within the community were recruited to deliver the interventions. After qualifying for the job based on a written and oral exam, those who were accepted then attended a week long residential training session. The training sessions included basic training and component-specific training, as well as refresher training on a quarterly basis. Training programmes were interactive, included practice sessions and role play, and addressed technical components of the intervention, communication and negotiation skills. Community health promoters were instructed to make weekly visits for first 6 months, followed by fortnightly visits to intervention households, and were paid a monthly stipend (equivalent to US$20).

The intervention was based on the Integrated Behavioural Model for Water Sanitation and Hygiene, addressing contextual, psychosocial, and technology factors at the societal and structural, community, interpersonal, individual, and habitual level.^14^ Key messages were delivered through Behaviour Change Communication materials—eg, visual aids including flip charts, posters, and reminder cue cards; interactive activities with songs and games; and the distribution of study group-specific hardware, products, or supplements.

The water treatment group was provided with a 10 L vessel with a lid, tap, and regular supply of 33 mg sodium dichloroisocyanurate tablets (Medentech, Wexford, Ireland) per 10 L drinking water. We did spot checks for residual chlorine in the water treatment group from supplied water storage container by using a HACH colourimeter.

The sanitation intervention was delivered at the compound level; compounds include rural households where patrilineally linked families live, which are usually arranged around a common courtyard. For all latrines in the compound that did not have a slab, a functional water seal or construction that prevented surface runoff of a faecal stream into the community were decommissioned and replaced. If the index household did not have their own latrine, the project built a latrine; the project also upgraded latrines for all the compound households that had an unhygienic latrine. The standard project intervention latrine was a double pit latrine with a water seal. The project also provided potties to children younger than 3 years and a sani-scoop (a spade-like hand tool) for removing faeces from the compound.

The handwashing treatment group received two handwashing stations per index household, one with a 40 L water reservoir placed near the latrine and a 16 L reservoir for the kitchen. Each handwashing station included a basin to collect rinse water and a bottle with a regular supply of detergent sachets for making soapy water.

The nutrition treatment group received a regular supply of lipid-based nutrient supplements (Nutriset, Malaunay, France) for children aged 6–24 months. Promoters instructed caregivers to feed a 10 g nutrient sachet (118 kcal, 9·6 g fat, 2·6 g protein, 12 vitamins, and ten minerals) to the index child twice daily; breastfeeding and complementary feeding intervention messages were adapted from the Alive and Thrive program in Bangladesh.^15^ Nutrition messages were focused on maternal extra food intake, dietary diversity, early initiation of colostrum within 30 min of the delivery, breastfeeding techniques (positioning and attachment), exclusive breastfeeding up to 6 months, and timely initiation of complementary feeding along with breastfeeding (6–24 months). Messages also included instructions about complementary food preparation along with serving portions and instructions about promoting children's self-feeding. By contrast, control children in the study were getting no such specific nutritional interventions or promoters visits. However, they might have received child feeding information (eg, about colostrum, breastfeeding, or complementary feeding) from government health workers during health visits or antenatal checkup as per national guidelines for infant and young child feeding in Bangladesh.

After enrolment, baseline information was collected using standard questionnaires on the socioeconomic and demographic status of the participants, including parental education, maternal age, number of children younger than 18 years in household, total number of people in the compound, household assets and land ownership, homestead, water, sanitation, and hygiene conditions, behaviour of the household members, household food insecurity; and household structure (floor construction). At the baseline, year 1, and year 2 surveys, data were collected on intervention quality (chlorine spot check, compliance, child faeces disposal, presence of handwashing stations with soap and water, consumption rate of lipid-based nutrient supplements, and so on), as well as on maternal and child outcome measures, including health and hygiene information, morbidity, nutritional and developmental outcomes, and other biochemical measures.

### Outcomes

Here we report on the prespecified secondary child development outcomes of the trial.^13^ When children were aged about 1 year (range 7–17 months), we assessed gross motor milestones using the WHO module, which consists of direct assessments and parental reports of whether their child can perform certain actions (eg, standing with support).^16^ We also assessed language development (understanding and speaking words) using the MacArthur-Bates Communicative Development Inventories, which collects information about child language development via parental report; these well-established measures are validated for use in Bangladesh.^17^

When children were aged about 2 years (range 21–30 months), we assessed communication, gross motor, and personal social development using the Extended Ages and Stages Questionnaire (EASQ), which is mainly a parental report that we adapted for use in low-income and middle-income countries,^18^ using standard techniques.^19^ During adaptation, some direct tests (about 25% of total items) were added for behaviours that parents might fail to observe—eg, pointing at pictures in the picture book, naming body parts, kicking ball, offering toys to own mirror image, copying gestures (appendix). The domains of the tests were arranged in 2–3 month age bands with three responses: yes, sometimes, or not yet. To create the reference distributions for the communication, gross motor, and personal social subscales and the overall global scale of the EASQ, the summed age-specific raw scores from the control group were standardised with a mean of 0 and a SD of 1, yielding *Z* scores for each 2-month age band. Standardised *Z* scores for the rest of the sample were then created using the reference distribution for each age band.

At age 2 years, we used two executive function tests—the A-not-B task and the Tower test—to directly assess children's impulse control, ability to initiate action, ability to sustain attention, and their persistence. The A-not-B task focused on working memory of children and was recently adapted and standardised in Bangladesh for young children.^20^ In this test a treat is hidden in front of a child in one of two shallow wells on a wooden board and covered with the two opaque cups; after distraction for 5 s, the child is asked to lift the right cup and get the treat. Sum of correct attempts out of ten trials gives the total score. The Tower test, where children took turns to make towers out of eight blocks with a tester (university graduate), was adapted after extensive piloting. In pilot tests on 77 children younger than 2 years, the Tower test showed good test–retest reliability (correlation coefficient r=0·73; p=0·02) and moderate concurrent validity, when measured by assessing correlation with the Family Care Indicator at the same time (r=0·21, p=0·05).

At ages 1 and 2 years, we collected information about behaviours related to responsive parenting (eg, activities and outings for children, toys and books available in the home), using items adapted from the Home Measurement for Observation for the Environment (HOME)^21^ and from the UNICEF Multi-Indicator Cluster Surveys.^22^ Maternal depressive symptoms were assessed at both timepoints using the Centers for Epidemiological Studies-Depression Scale (CESD), a brief, widely used measure of 20 statements that assess the likelihood of depressive symptomology; this tool has been previously culturally adapted and used in other studies set in Bangladesh.^23^

All tests of child development and maternal wellbeing were piloted on 77 non-study children aged 18–24 months and their mothers, and showed face validity, meaningful correlation with developmental measures, association with sociodemographic variables, and test–retest reliability at 7-day intervals (correlation coefficient r for all tests >0·70).

Eight testers (university graduates) received 5–10 days of extensive hands-on training, including theoretical and practical sessions. When agreement achieved at least 90% between testers and trainers, the interviewers were considered ready for the main study. For 5–10% of total tests we looked for ongoing reliability across the time period by comparing the testers with a trained supervisor (gold standard) who had a psychology background. We arranged refresher trainings every 6 months or when correlation coefficients (r) for any tester with a supervisor fell below 0·85.

### Statistical analysis

The trial was powered to detect a difference of 0·15 in the primary outcome length-for-age *Z* score (LA*Z*) in comparisons of intervention groups against control, accounting for repeated measures within clusters.^13^ This design allocated one third of clusters to intervention (for any single group comparison against control), assumed seven children per cluster, 80% power with a two-sided alpha of 0·05, and intra-cluster correlation of 0·05, and had a minimum detectable effect size for the EASQ *Z* scores of 0·16 using a standard equation for cluster randomised trials.^24^ Under the same assumptions, the minimum detectable effect for comparison of combined versus single intervention groups for the EASQ *Z* scores was 0·18.

All analyses were intention to treat. Because randomisation was geographically pair-matched in blocks of eight clusters, we estimated unadjusted mean differences using generalised linear models that considered pair matching and block-level clustering. The geographical pair-matching ensured that measurement timing was balanced across groups. The pair-matched analyses removed any potential confounding from seasonal changes in baseline risk. For each comparison, we estimated p values using a paired t-test and cluster-level means. Because the children were of wide age ranges and the tests are age dependent, we used age-specific z scores of tests, within 2 month age bands, and controlled for age in the analysis.

We summarised the distribution of continuous outcomes using Gaussian kernel density smoothers using default bandwidth and kernel selection in R. We also estimated adjusted mean differences by adjusting for baseline covariates that could be considered potential confounders: child sex, maternal age, maternal height (in cm), parents' education in year of schooling, number of children younger than 18 years in household, total number of people in the compound, food insecurity of household (measured using Household Hunger Scale), housing materials (construction materials and utilities), household assets, distance to water source, and month of measurement. For adjusted analyses, we only included the covariates that were associated (p<0·2) with the outcomes using a likelihood ratio test. We conducted pre-specified subgroup analyses to examine effect modification by child sex, maternal parity, age and education, household hunger score and socioeconomic status.

To compare attainment rates for each of the WHO motor milestones, we estimated hazard ratios from current status data using a semiparametric generalised additive model with complementary log-log link and baseline hazard fit with a monotonic cubic spline. All analyses were done with R (version 3.2.4) and STATA (version 13.0).

The trial is registered with ClinicalTrials.gov, NCT01590095.

### Role of the funding source

The funder reviewed and approved the experimental design, but was not involved in data collection, data analysis, data interpretation, or writing of the report. The corresponding author had full access to all of the data in the study and had final responsibility for the decision to submit for publication.

## Results

**Figure 1:**
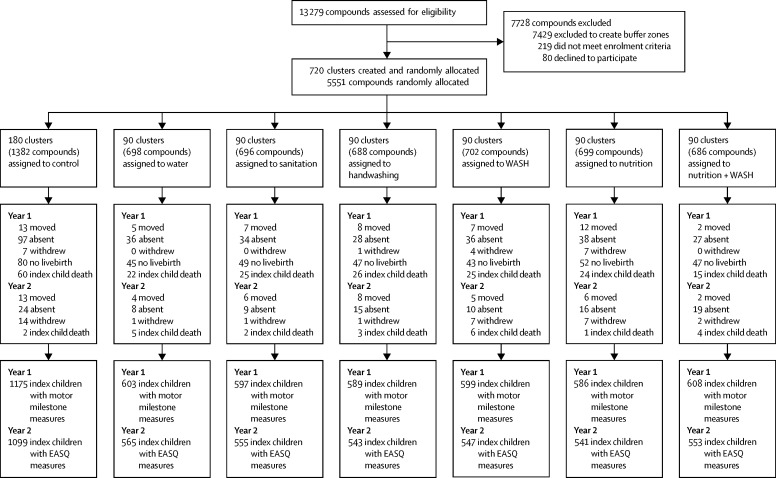
Trial profile Numbers are children except where specified. Attrition was only at the child level; no cluster dropped out. WASH=water, sanitation, and handwashing. EASQ=Extended Ages and Stages Questionnaire.

We identified 13 279 pregnant women in their first or second trimester. About half of these women were excluded to create 1 km buffer zones between intervention clusters. Between May 31, 2012, and July 7, 2013, we randomly allocated 720 clusters and enrolled 5551 women to one of the six intervention groups (90 clusters each) or to the control group (180 clusters; figure 1). Index children of 928 (17%) enrolled women were lost to follow-up in year 1 and an additional 201 (3%) women in year 2 for various reasons—eg, stillbirth (n=363 [6%]), death before the final assessment (n=220 [4%]), out migration (n=98 [2%]), absent on repeated follow-up (n=397 [7%]), or refusal (n=52 [<1%]). Losses to follow-up were balanced across groups (appendix). 4757 children were assessed at 1 year and 4403 at 2 years (figure 1). Treatment groups were well balanced on all baseline demographic characteristics, including maternal and paternal education, household composition, and household wealth; groups were also balanced with regards to drinking water source, sanitation practices and access to sanitation-related supplies, and handwashing supplies and practices (table 1). There was high adherence of all groups to the assigned interventions, with uptake of more than 80% in the single intervention groups and similar uptake in combined intervention groups (appendix).

**Table 1:** Baseline characteristics

		**Control group (N=1382)**	**Water group (N=698)**	**Sanitation group (N=696)**	**Handwashing group (N=688)**	**Combined water, sanitation, and handwashing group (N=702)**	**Nutrition (N=699)**	**Combined water, sanitation, handwashing, and nutrition group (N=686)**
**Maternal**								
Age (years)		23·6 (5·0)	23·7 (5·2)	23·7 (5·2)	23·8 (5·5)	24·3 (5·5)	23·7 (5·1)	23·8 (5·5)
Years of education		5·9 (3·4)	5·8 (3·4)	5·8 (3·5)	5·8 (3·3)	5·9 (3·3)	5·8 (3·5)	5·6 (3·5)
**Paternal**								
Years of education		4·9 (4·0)	4·9 (4·1)	5·0 (4·2)	4·6 (4·1)	5·0 (4·2)	4·8 (4·0)	4·7 (3·9)
Works in agriculture		414 (30%)	224 (32%)	204 (29%)	249 (36%)	216 (31%)	232 (33%)	207 (30%)
**Household**								
Number of people		4·7 (2·3)	4·6 (2·2)	4·7 (2·1)	4·7 (2·2)	4·7 (2·1)	4·7 (2·2)	4·7 (2·1)
Has electricity		784 (57%)	422 (60%)	408 (59%)	405 (59%)	426 (61%)	409 (59%)	412 (60%)
Has a cement floor		145 (10%)	82 (12%)	85 (12%)	55 (8%)	77 (11%)	67 (10%)	72 (10%)
Acres of agricultural land owned		0·15 (0·21)	0·14 (0·20)	0·14 (0·22)	0·14 (0·20)	0·15 (0·23)	0·16 (0·27)	0·14 (0·38)
**Drinking water**								
Tubewell primary water source		1038 (75%)	500 (72%)	519 (75%)	482 (70%)	546 (78%)	519 (74%)	504 (73%)
Stored water observed at home		666 (48%)	353 (51%)	341 (49%)	347 (50%)	304 (43%)	301 (43%)	331 (48%)
**Sanitation**								
Daily defecation in the open								
	Adult men	97 (7%)	39 (6%)	52 (8%)	64 (9%)	54 (8%)	59 (9%)	50 (7%)
	Adult women	62 (4%)	18 (3%)	33 (5%)	31 (5%)	29 (4%)	39 (6%)	24 (4%)
	Children aged 8 to <15 years	53 (10%)	25 (9%)	28 (9%)	43 (15%)	30 (10%)	23 (8%)	28 (10%)
	Children aged 3 to <8 years	267 (38%)	141 (37%)	137 (38%)	137 (39%)	137 (38%)	129 (39%)	134 (37%)
	Children aged 0 to <3 years	245 (82%)	112 (85%)	117 (84%)	120 (85%)	123 (79%)	128 (85%)	123 (88%)
Latrine								
	Owned	750 (54%)	363 (52%)	374 (54%)	372 (54%)	373 (53%)	377 (54%)	367 (53%)
	Concrete slab	1251 (95%)	644 (95%)	610 (92%)	613 (93%)	620 (93%)	620 (94%)	621 (94%)
	Functional water seal	358 (31%)	183 (31%)	177 (30%)	162 (28%)	152 (26%)	183 (31%)	155 (27%)
	Visible stool on slab or floor	625 (48%)	350 (53%)	332 (52%)	335 (52%)	289 (44%)	331 (51%)	298 (46%)
	Owned a potty	61 (4%)	27 (4%)	28 (4%)	35 (5%)	27 (4%)	36 (5%)	30 (4%)
Human faeces observed								
	In the house	114 (8%)	65 (9%)	56 (8%)	70 (10%)	48 (7%)	58 (8%)	49 (7%)
	In child's play area	21 (2%)	6 (1%)	6 (1%)	8 (1%)	7 (1%)	8 (1%)	7 (1%)
**Handwashing**								
Within six steps of latrine								
	Has water	178 (14%)	83 (13%)	81 (13%)	63 (10%)	67 (10%)	62 (10%)	72 (11%)
	Has soap	88 (7%)	50 (8%)	48 (8%)	34 (5%)	42 (7%)	32 (5%)	36 (6%)
Within six steps of kitchen								
	Has water	118 (9%)	51 (8%)	51 (8%)	45 (7%)	61 (9%)	61 (9%)	60 (9%)
	Has soap	33 (3%)	18 (3%)	14 (2%)	13 (2%)	15 (2%)	23 (3%)	18 (3%)
**Nutrition**								
Household is food secure*		932 (67%)	495 (71%)	475 (68%)	475 (69%)	482 (69%)	479 (69%)	485 (71%)

Data are n (%) or mean (SD). Percentages were estimated from slightly smaller denominators than those shown at the top of the table for the following variables due to missing values: father works in agriculture, open defecation, latrine has a concrete slab, latrine has a functional water seal, visible stool on latrine slab or floor, ownership of child potty, observed faeces in the house or child's play area, handwashing variables.

* Assessed by the Household Food Insecurity Access Scale.^25^

At 1 year follow-up, the median age of the children was 11 months (IQR 10–13). The age of attainment of each of the motor milestones in the study group was slightly delayed compared with the WHO reference population (table 2). There were some improvements in motor milestone attainment for children in the nutrition group or the combined water, sanitation, handwashing, and nutrition group (table 3). Specifically, compared with the control group, the combined water, sanitation, handwashing, and nutrition group had a greater rate of attaining the standing alone milestone (conditional on not standing upright on both for 10 s; hazard ratio [HR], 95% CI 1·01–1·40) and the nutrition group had a greater rate of attaining the walking alone milestone (conditional on not walking independently for 5 steps; 1·32, 1·07–1·62). The rate of attaining the walking alone milestone was higher in the combined water, sanitation, handwashing, and nutrition group than in the combined group without nutrition (HR 1·29, 95% CI 1 ·01–1·65). These improvements were robust to the inclusion of covariates in adjusted analyses (appendix).

**Table 2:** Estimated age of attainment for each of the motor milestones among children in the study population compared with the WHO reference population

	WHO growth standards reference*, **median (IQR) age of attainment, months**	**WASH Benefits study children**†, **median (IQR) age of attainment, months**	**Age 7–9 months (N=282)**	**Age 9–11 months (N=1603)**	**Age 11–13 months (N=1989)**	**Age 13–15 months (N=843)**
Sitting without support	5·9 (5·8–6·0)	··	280 (99·6%)	1588 (99·3%)	1979 (99·5%)	835 (99·3%)
Standing with assistance	7·4 (6·6–8·4)	8·7 (8·0–9·3)	141 (50·5%)	1320 (83·1%)	1860 (94·5%)	814 (97·4%)
Hands-and-knees crawling	8·3 (8·2–8·4)	··	162 (57·7%)	1111 (69·5%)	1504 (76·0%)	672 (80·0%)
Walking with assistance	9·0 (8·2–10·0)	10·5 (9·1–11·3)	29 (10·4%)	739 (46·4%)	1579 (80·3%)	760 (91·1%)
Standing alone	10·8 (9·7–12·0)	11·9 (10·9–13·7)	6 (2·1%)	237 (14·9%)	965 (48·9%)	631 (75·8%)
Walking alone	12·0 (11·0–13·0)	13·0 (11·9–14·1)	1 (0·4%)	47 (2·9%)	527 (26·7%)	529 (63·3%)

* Published data from the WHO Multicentre Growth Reference Study.^14^

† Estimated using non-parametric maximum likelihood estimator of cumulative probability of attainment. Median age could not be estimated for the sitting without support and hands-and-knees crawling milestones because >80% of children had already achieved this milestone before the assessment.

**Table 3:** Relative rate of motor milestone attainment after 1 year of intervention

	**N**	**Hazard ratio *vs* control group (95% CI)**	**Hazard ratio *vs* combined water, sanitation, and handwashing group group (95% CI)**	**Hazard ratio *vs* nutrition group (95% CI)**
**Standing with assistance**				
Control	1161	1 (ref)	··	··
Water	598	0·99 (0·86–1·14)	··	··
Sanitation	593	1·03 (0·89–1·19)	··	··
Handwashing	584	0·90 (0·78–1·03)	··	··
Combined water, sanitation, handwashing	594	0·95 (0·83–1·09)	1 (ref)	··
Nutrition	580	1·13 (0·98–1·31)	··	1 (ref)
Combined water, sanitation, handwashing, and nutrition	603	1·05 (0·91–1·20)	1·11 (0·95–1·30)	0·94 (0·80–1·10)
**Hands-and-knees crawling**				
Control	1171	1 (ref)	··	··
Water	601	0·94 (0·83–1·06)	··	··
Sanitation	593	0·98 (0·86–1·11)	··	··
Handwashing	589	0·85 (0·75–0·96)	··	··
Combined water, sanitation, handwashing	595	0·91 (0·80–1·03)	1 (ref)	··
Nutrition	584	1·02 (0·90–1·15)	··	1 (ref)
Combined water, sanitation, handwashing, and nutrition	606	0·98 (0·87–1·11)	1·08 (0·94–1·25)	0·96 (0·84–1·11)
**Walking with assistance**				
Control	1160	1 (ref)	··	··
Water	598	1·06 (0·93–1·21)	··	··
Sanitation	591	1·09 (0·95–1·25)	··	··
Handwashing	583	0·87 (0·76–1·01)	··	··
Combined water, sanitation, handwashing	594	0·98 (0·85–1·13)	1 (ref)	··
Nutrition	580	1·12 (0·98–1·29)	··	1 (ref)
Combined water, sanitation, handwashing and nutrition	604	0·99 (0·87–1·14)	1·02 (0·87–1·19)	0·88 (0·75–1·03)
**Standing alone**				
Control	1165	1 (ref)	··	··
Water	600	1·14 (0·97–1·34)	··	··
Sanitation	591	1·10 (0·93–1·29)	··	··
Handwashing	585	0·96 (0·81–1·14)	··	··
Combined water, sanitation, handwashing	592	1·02 (0·86–1·21)	1 (ref)	··
Nutrition	582	1·14 (0·96–1·34)	··	1 (ref)
Combined water, sanitation, handwashing, and nutrition	607	1·19 (1·01–1·40)	1·17 (0·97–1·41)	1·03 (0·86–1·25)
**Walking alone**				
Control	1165	1 (ref)	··	··
Water	600	1·26 (1·03–1·54)	··	··
Sanitation	592	1·15 (0·93–1·41)	··	··
Handwashing	585	1·09 (0·87–1·35)	··	··
Combined water, sanitation, handwashing	593	0·94 (0·75–1·17)	1 (ref)	··
Nutrition	581	1·32 (1·07–1·62)	··	1 (ref)
Combined water, sanitation, handwashing, and nutrition	607	1·21 (0·98–1·48)	1·29 (1·01–1·65)	0·90 (0·72–1·14)

**Figure 2:**
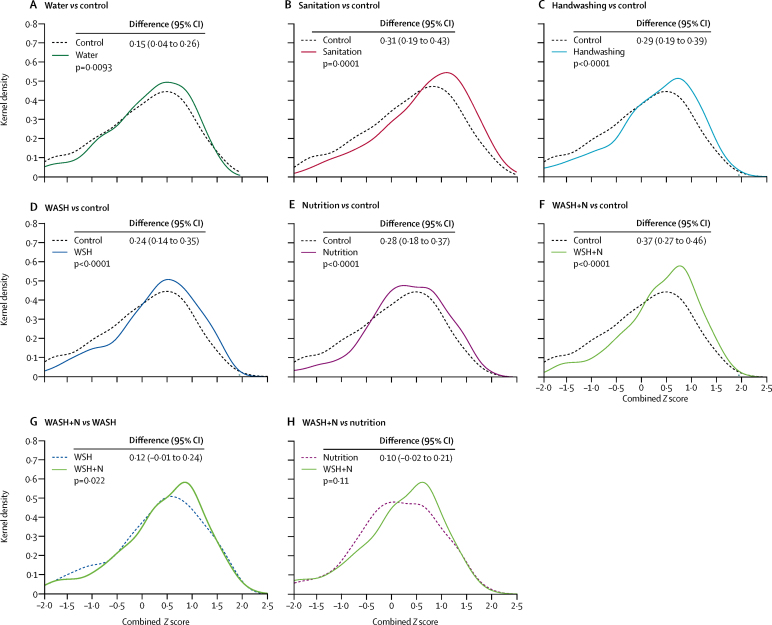
Kernal density plots of combined *Z* scores of the Extended Ages and Stages Questionnaire at year 2 follow-up Kernel density plots summarise the distribution of combined *Z* scores of index children who were born into the study and were between 21–30 months (median 26 months, IQR 24–27) at the time of measurement. Data are mean difference (95% CI). WASH=water, sanitation, and handwashing. N=nutrition.

At year 2 follow-up, the median age of children was 26 months (IQR 24–27). There were consistent beneficial effects of the intervention on all sub-scales of the EASQ child development measure (communication, gross motor, personal social), with largest effects in the combined water, sanitation, handwashing, and nutrition group (table 4, figure 2, appendix). Compared with the control group, all intervention groups except the water treatment group had higher scores on the communication and gross motor subscales, and all intervention groups had higher scores on the personal social subscale (table 4). In the combined EASQ measure including all subscales, effect sizes were smallest in the water treatment group (mean difference 0·15, 95% CI 0·04–0·26 compared with control) and largest in the combined water, sanitation, handwashing, and nutrition group (0·37, 0·27–0·46; figure 2). The improvements were robust to the inclusion of covariates in adjusted analyses (appendix).

**Table 4:** Standardised differences in scores on the communication, gross motor, personal social, and combined scales of the Extended Ages and Stages Questionnaire after 2 years of intervention

	**N, mean (SD)**	**Mean difference *vs* control group (95% CI)**	**Mean difference *vs* combined water, sanitation, and handwashing group (95% CI)**	**Mean difference *vs* nutrition group (95% CI)**
**Communication** Z **score**				
Control	1099, 0·00 (1·00)	0 (ref)	··	··
Water	565, 0·09 (0·96)	0·10 (−0·02 to 0·21)	··	··
Sanitation	555, 0·20 (0·95)	0·21 (0·09 to 0·33)	··	··
Handwashing	543, 0·20 (0·94)	0·21 (0·10 to 0·31)	··	··
Combined water, sanitation, and handwashing	547, 0·15 (0·93)	0·14 (0·03 to 0·26)	0 (ref)	··
Nutrition	541, 0·19 (0·86)	0·19 (0·10 to 0·28)	··	0 (ref)
Combined water, sanitation, handwashing, and nutrition	553, 0·25 (0·90)	0·26 (0·16 to 0·36)	0·11 (−0·02 to 0·25)	0·07 (−0·05 to 0·19)
**Gross motor** Z **score**				
Control	1099, 0·00 (1·00)	0 (ref)	··	··
Water	557, 0·01 (0·93)	0·01 (−0·11 to 0·13)	··	··
Sanitation	549, 0·12 (0·93)	0·12 (0·00 to 0·25)	··	··
Handwashing	535, 0·10 (0·93)	0·12 (0·00 to 0·23)	··	··
Combined water, sanitation, and handwashing	539, 0·16 (0·87)	0·16 (0·04 to 0·27)	0 (ref)	··
Nutrition	528, 0·18 (0·95)	0·19 (0·08 to 0·30)	··	0 (ref)
Combined water, sanitation, handwashing, and nutrition	546, 0·14 (0·94)	0·14 (0·03 to 0·25)	−0·01 (−0·12 to 0·11)	−0·05 (−0·19 to 0·10)
**Personal social** Z **score**				
Control	1099, 0·00 (1·00)	0 (ref)	··	··
Water	557, 0·11 (0·91)	0·13 (0·01 to 0·25)	··	··
Sanitation	544, 0·28 (0·97)	0·29 (0·18 to 0·40)	··	··
Handwashing	528, 0·26 (0·94)	0·28 (0·17 to 0·40)	··	··
Combined water, sanitation, and handwashing	538, 0·28 (1·01)	0·27 (0·16 to 0·38)	0 (ref)	··
Nutrition	528, 0·22 (0·97)	0·22 (0·11 to 0·33)	··	0 (ref)
Combined water, sanitation, handwashing, and nutrition	538, 0·34 (0·98)	0·35 (0·24 to 0·46)	0·07 (−0·06 to 0·20)	0·13 (−0·01 to 0·28)
**Combined** Z **score**				
Control	1099, 0·00 (1·00)	0 (ref)	··	··
Water	539, 0·14 (0·86)	0·15 (0·04 to 0·26)	··	··
Sanitation	531, 0·31 (0·86)	0·31 (0·19 to 0·43)	··	··
Handwashing	502, 0·27 (0·87)	0·29 (0·19 to 0·39)	··	··
Combined water, sanitation, and handwashing	519, 0·25 (0·90)	0·24 (0·14 to 0·35)	0 (ref)	··
Nutrition	506, 0·27 (0·83)	0·28 (0·18 to 0·37)	··	0 (ref)
Combined water, sanitation, handwashing, and nutrition	522, 0·36 (0·81)	0·37 (0·27 to 0·46)	0·12 (−0·01 to 0·24)	0·10 (−0·02 to 0·21)

We noted benefits in the comprehension subscale of the MacArthur Communicative Development Inventories for the water treatment group, handwashing group, and in both combined intervention groups in year 1 (appendix), as well as in both the comprehension and the expressive language subscales for all intervention groups in year 2 (table 5). However, the effect sizes were not consistently significantly different across the intervention groups. There were no effects of any intervention on executive function measures (table 5).

**Table 5:** Effect of the interventions on communication and executive functions after 2 years

		**N, mean (SD)**	**Mean difference *vs* control group (95% CI)**	**Mean difference *vs* combined water, sanitation, and handwashing group (95% CI)**	**Mean difference *vs* nutrition group (95% CI)**
**MacArthur-Bates Communicative Development Inventories**					
Comprehension score					
	Control	1106, 0·00 (1·00)	0 (ref)	··	··
	Water	535, 0·18 (0·88)	0·20 (0·08 to 0·32)	··	··
	Sanitation	524, 0·16 (0·87)	0·18 (0·05 to 0·31)	··	··
	Handwashing	519, 0·21 (0·80)	0·22 (0·11 to 0·33)	··	··
	Water, sanitation, and handwashing	513, 0·18 (0·85)	0·18 (0·07 to 0·29)	0 (ref)	··
	Nutrition	501, 0·19 (0·80)	0·19 (0·08 to 0·29)	··	0 (ref)
	Water, sanitation, handwashing, and nutrition	528, 0·25 (0·86)	0·26 (0·14 to 0·38)	0·07 (−0·05 to 0·19)	0·07 (−0·05 to 0·20)
Expressive language score					
	Control	1106, 0·00 (1·00)	0 (ref)	··	··
	Water	497, 0·18 (0·88)	0·18 (0·07 to 0·30)	··	··
	Sanitation	473, 0·17 (0·90)	0·17 (0·06 to 0·29)	··	··
	Handwashing	472, 0·19 (0·86)	0·19 (0·09 to 0·30)	··	··
	Water, sanitation, and handwashing	470, 0·13 (0·97)	0·11 (0·00 to 0·23)	0 (ref)	··
	Nutrition	461, 0·19 (0·86)	0·18 (0·07 to 0·29)	··	0 (ref)
	Water, sanitation, handwashing, and nutrition	484, 0·19 (0·92)	0·20 (0·08 to 0·32)	0·07 (−0·07 to 0·22)	0·01 (−0·14 to 0·16)
**Executive function**					
Tower test *Z* score					
	Control	1106, 0·00 (1·00)	0 (ref)	··	··
	Water	582, 0·09 (0·99)	0·09 (−0·01 to 0·20)	··	··
	Sanitation	564, 0·01 (1·00)	0·02 (−0·11 to 0·15)	··	··
	Handwashing	559, 0·12 (0·90)	0·13 (0·02 to 0·24)	··	··
	Water, sanitation, and handwashing	566, 0·06 (1·01)	0·06 (−0·05 to 0·18)	0 (ref)	··
	Nutrition	550, 0·07 (0·94)	0·07 (−0·04 to 0·18)	··	0 (ref)
	Water, sanitation, handwashing, and nutrition	571, 0·11 (0·95)	0·10 (−0·01 to 0·22)	0·03 (−0·11 to 0·16)	0·02 (−0·12 to 0·15)
A-not-B test *Z* score					
	Control	1106, 0·00 (1·00)	0 (ref)	··	··
	Water	579, 0·17 (0·88)	0·17 (0·06 to 0·28)	··	··
	Sanitation	560, 0·10 (0·92)	0·10 (−0·01 to 0·20)	··	··
	Handwashing	555, 0·08 (0·94)	0·08 (−0·02 to 0·18)	··	··
	Water, sanitation, and handwashing	565, 0·05 (0·98)	0·05 (−0·05 to 0·16)	0 (ref)	··
	Nutrition	545, 0·04 (0·95)	0·04 (−0·06 to 0·14)	··	0 (ref)
	Water, sanitation, handwashing, and nutrition	568, 0·08 (0·93)	0·07 (−0·01 to 0·16)	0·01 (−0·10 to 0·13)	0·03 (−0·07 to 0·14)

Measures of maternal depressive symptoms were lower in all intervention groups than the control group at both year 1 and year 2 (appendix). Compared with the control group, responsive parenting assessed by the HOME scales was improved at year 1 in the sanitation group, handwashing group, and combined water, sanitation, handwashing, and nutrition group), and at year 2 in the water treatment group, nutrition group, and combined water, sanitation, handwashing, and nutrition group (appendix).

We found no consistent evidence for effect modification by child sex; maternal parity, age, education, household hunger score, and socioeconomic status in additional pre-specified subgroup analyses (appendix).

## Discussion

In this trial of independent and combined water, sanitation, handwashing, and nutritional interventions provided to households in rural Bangladesh, we found some benefits to children after 1 year of exposure when they were about 12 months old. We also found consistent developmental benefits across a range of outcomes among children in all intervention groups after 2 years of exposure when children were approximately 24 months old; there were no significant effects on measures of executive function. Rates of motor milestone attainment at age 1 year were faster in children in the combined water, sanitation, handwashing, and nutrition group and in the nutrition alone group. Benefits in domain-specific developmental outcomes (communication, gross motor, and personal social subscales of the EASQ; MacArthur-Bates comprehension and expressive language) measured at age 2 years were more consistent, and were apparent in almost all intervention groups; the only domain in which there was no consistent effect was executive function at this age.

The distribution plots suggest some potential ceiling effects in the MacArthur-Bates communication (both comprehensive and expressive language) scores in year 1 but not in year 2. This finding could be due to reporting bias of mothers about their children's early abilities, which reduced with progression of age in year 2 (appendix). The two executive tests we used were possibly slightly difficult for these 2-year-old Bangladeshi children. Although executive function skills begin to develop shortly after birth, different tests to measure these abilities suggest that they are best assessed between 3 to 5 years of age, when substantial brain growth in areas responsible for these skills occurs.^26^ For language, gross motor, personal social, and all communication domains, effect sizes ranged from 0·13 to 0·35 SDs.

Several development-specific and development-sensitive mechanisms might have played a role in affecting children's development in our study.^27^ Possible mechanisms linking improved water, sanitation, and handwashing practices with better developmental outcomes include reduced infection, reduced inflammation, and increased social interaction, all of which could improve synaptic connections as well as myelination in the CNS, thus benefitting developmental outcomes.^28^ A main link connecting water, sanitation, and handwashing interventions with child development is care practices of parents and other adult caregivers, relating to feeding, nutrition, and health, which can have direct effects on child outcomes.^29^ However, the improved developmental outcomes could also have resulted from an indirect pathway by improving maternal wellbeing and reducing maternal stress or depression. If the interventions themselves change a mother's ability to provide support and nurturing care for her children, maternal depression may connect water, sanitation, and handwashing interventions and improved cognitive outcomes.^30^ We found clear effects of all intervention groups on reducing maternal depressive symptoms, and some evidence for an improved home environment, suggesting that this pathway might be operating here. One of our key hypotheses for why the interventions were effective is that families received frequent visits and support from community health workers in all intervention groups.

Another clear pathway connecting water, sanitation, and handwashing interventions with developmental outcomes is via nutrition, particularly through linear growth faltering (low height-for-age or stunting);^31^ however, this pathway is unlikely to have affected our results, given that the water, sanitation, and handwashing interventions—alone or in combination—did not affect linear growth.^11^ In the nutrition group, which included lipid-based nutrient supplements and nutritional messages, there were improvements on stunting and wasting in index children,^11^ suggesting that some effects on development could occur via growth, or directly from the lipid-based nutrient supplement formulation. In a recent study evaluating prenatal and postnatal lipid-based nutrient supplements and micronutrient powders,^20^ lipid-based nutrient supplements had positive effects on motor and language development, but no effects on personal social behaviour or executive function. Similarly, a study in Burkina Faso^32^ also reported significant benefits of lipid-based nutrient supplements on children's motor, language, and personal social development, and a trial in Ghana^33^ found significant beneficial effects of lipid-based nutrient supplements on children's development. By contrast, there was no benefit of lipid-based nutrient supplements on child development measures in Malawian children given the supplement at 6–18 months of age.^34^

Another pathway that potentially links water, sanitation, and handwashing interventions with developmental outcomes is via enteric infections, including intestinal worms caused by poor sanitation, which can then result in iron deficiency and have negative consequences for child development.^35^ Experiencing repeated and prolonged episodes of diarrhoea could have direct effects on cognitive outcomes.^36^ In our trial, children receiving sanitation, handwashing, nutrition, and all combined interventions had roughly 40% lower diarrhoea prevalence compared with control.^11^ The intervention groups might also have reduced systemic inflammation (eg, CRP, sCD14, IL-1β and IL6), which has been associated with improved neurodevelopmental outcomes,^37^ and this pathway may also be operating here. Assessment is currently underway to investigate this potential pathway.^13^

In the parallel WASH Benefits study in rural Kenya, there were few developmental benefits despite similar types of interventions and similar growth benefits in the two nutrition groups.^10^, ^12^ This discrepancy could be due to less intense contact between health promoters with respondents (one contact per month in Kenya *vs* up to six contacts per month in Bangladesh), leading to less uptake of the targeted behaviours. An important area for future research is to establish whether similar effects, as found in Bangladesh, can be achieved with a frequency of promoter contacts that could be supported in large-scale interventions.

The main strengths of this study include the pair-matched cluster-randomised design, large sample size, high intervention adherence (as determined by objective indicators^11^), intensive interventions (often weekly or more frequently), and the use of multiple direct and indirect developmental indicators that were locally adapted to Bangladeshi children. Data collectors were rigorously trained for at least 1 week and showed high inter-rater agreement and test–retest reliabilities before the start of data collection. Ongoing quality assurance was also monitored. The consistent pattern of benefits in most of the domains of development across intervention groups further strengthens the internal consistency of the results.

Despite these strengths, the inferences that we can draw from this study are limited by some features of the design. One limitation is that we cannot know how much benefit resulted from social interaction and how much from reduced disease, reduced inflammation, or improved nutrition. Interventions delivered at the household level were intense (delivered weekly for the first 6 months and then fortnightly for next 18 months) and started from the second trimester of pregnancy. Thus, future research of this size and magnitude should explore direct and indirect effects of the interventions on outcomes, accounting for mediation by maternal depression, indicators of resources at home such as toys and books, and parental behaviours, along with active and passive control groups to account for contact with community health workers. Some of the questions answered by parental report (eg, MacArthur-Bates Communicative Development Inventories or some of the EASQ) might have been affected by courtesy bias, and in our study the parents and data collectors were not masked. To minimise the possibility of courtesy bias, we used a non-traditional approach to develop promoter skills that focused on collaborative problem-solving, rather than forceful advice-giving. Also, the assessment team was different from promoter team; the assessment team was unaware of the intervention details, joined the study midway, and only visited the families once a year to collect developmental data. Another limitation of our study is that effect estimates for single versus combined interventions fell slightly below the minimum detectable effect given the design, and thus were not statistically significant. Finally, it is unclear what predictive validity these early tests of child development might have for longer-term improvements in the trajectory of children's intellectual development. Thus, an important next step would be to follow up the children for longer to assess their development over time, especially into the pre-school and schooling years using direct child assessment measures.

In conclusion, our findings suggest that interventions designed to improve water quality, sanitation, handwashing practices, or nutrition have cumulative beneficial effects on children's developmental outcomes and go beyond the traditionally measured outcomes of growth and acute illness. Although some of our findings suggest that a combined intervention might have advantages over the individual interventions, we also found benefits of the single interventions in many cases, which would be a cheaper alternative to delivering combined interventions. Finally, given that this trial was a test of efficacy, an important next step would be to consider how to bring these interventions to scale—both individually and combined—with a focus on optimising intervention effectiveness and cost-effectiveness. Key learnings from the Scaling Up Nutrition movement, for example, have been that larger scale-up efforts require a clear vision for change, an enabling contextual environment (eg, household, community, political), actors, stakeholders and champions at multiple levels, government ownership, multiple incentives, adequate financial resources, and frameworks for monitoring, evaluation, learning, and accountability.^38^

## References

[1] Black MM, Walker SP, Fernald LCH (2017). Early childhood development coming of age: science through the life course. Lancet.

[2] Britto PR, Lye SJ, Proulx K, Early Childhood Development Interventions Review Group, for the Lancet Early Childhood development Series Steering Committee (2017). Nurturing care: promoting early childhood development. Lancet.

[3] Bhutta ZA, Ahmed T, Black RE (2008). What works? Interventions for maternal and child undernutrition and survival. Lancet.

[4] Ruel MT, Alderman H, Group MaCNS (2013). Nutrition-sensitive interventions and programmes: how can they help to accelerate progress in improving maternal and child nutrition?. Lancet.

[5] Wolf J, Pruss-Ustun A, Cumming O (2014). Assessing the impact of drinking water and sanitation on diarrhoeal disease in low- and middle-income settings: systematic review and meta-regression. Trop Med Int Health.

[6] Dangour AD, Watson L, Cumming O (2013). Interventions to improve water quality and supply, sanitation and hygiene practices, and their effects on the nutritional status of children. Cochrane Database Syst Rev.

[7] MacIntyre J, McTaggart J, Guerrant RL, Goldfarb DM (2014). Early childhood diarrhoeal diseases and cognition: are we missing the rest of the iceberg?. Paediatr Int Child Health.

[8] Dearden KA, Brennan AT, Behrman JR (2017). Does household access to improved water and sanitation in infancy and childhood predict better vocabulary test performance in Ethiopian, Indian, Peruvian and Vietnamese cohort studies?. BMJ Open.

[9] Bowen A, Agboatwalla M, Luby S, Tobery T, Ayers T, Hoekstra RM (2012). Association between intensive handwashing promotion and child development in Karachi, Pakistan: a cluster randomized controlled trial. Arch Pediatr Adolesc Med.

[10] Stewart CP, Kariger P, Pickering AJ (2018). Effects of water, sanitation, handwashing, and nutritional interventions on child development: a cluster-randomised controlled trial in rural Kenya. Lancet Child Adolesc Health.

[11] Luby SP, Rahman M, Arnold BF (2018). Effects of water quality, sanitation, handwashing, and nutritional interventions on diarrhoea and child growth in rural Bangladesh: a cluster randomised trial. Lancet Glob Health.

[12] Null C, Stewart CP, Pickering AJ (2018). Effects of water quality, sanitation, handwashing, and nutritional interventions on diarrhoea and child growth in rural Kenya: a cluster-randomised controlled trial. Lancet Glob Health.

[13] Arnold BF, Null C, Luby SP (2013). Cluster-randomised controlled trials of individual and combined water, sanitation, hygiene and nutritional interventions in rural Bangladesh and Kenya: the WASH Benefits study design and rationale. BMJ Open.

[14] Dreibelbis R, Winch PJ, Leontsini E (2013). The Integrated Behavioural Model for Water, Sanitation, and Hygiene: a systematic review of behavioural models and a framework for designing and evaluating behaviour change interventions in infrastructure-restricted settings. BMC Public Health.

[15] Menon P, Nguyen PH, Saha KK (2016). Combining intensive counseling by frontline workers with a nationwide mass media campaign has large differential impacts on complementary feedingpractices but not on child growth: results of a cluster-randomized program evaluation in Bangladesh. J Nutr.

[16] WHO Multicentre Growth Reference Study Group (2006). WHO Motor Development Study: windows of achievement for six gross motor development milestones. Acta Paediatri Suppl.

[17] Hamadani JD, Baker-Henningham H, Tofail F, Mehrin F, Huda SN, Grantham-McGregor SM (2010). Validity and reliability of mothers' reports of language development in 1-year-old children in a large-scale survey in Bangladesh. Food Nutr Bull.

[18] Fernald LCH, Kariger PK, Hidrobo M, Gertler PJ (2012). Socio-economic gradients in child development in very young children: evidence from India, Indonesia, Peru and Senegal. Proc Natl Acad Sci USA.

[19] Fernald LCH, Prado E, Kariger P, Raikes A (2017). A toolkit for measuring early childhood development in low- and middle-income countries. https://openknowledge.worldbank.org/bitstream/handle/10986/29000/WB-SIEF-ECD-MEASUREMENT-TOOLKIT.pdf?sequence=1&isAllowed=y.

[20] Matias SL, Mridha MK, Tofail F (2017). Home fortification during the first 1000 d improves child development in Bangladesh: a cluster-randomized effectiveness trial. Am J Clin Nutr.

[21] Bradley RH, Corwyn RF, McAdoo HP, Garcia Coll C (2001). The home environments of children in the United States Part I: variations by age, ethnicity, and poverty status. Child Dev.

[22] Kariger P, Frongillo EA, Engle P, Britto PM, Sywulka SM, Menon P (2012). Indicators of family care for development for use in multicountry surveys. J Health Popul Nutr.

[23] Black MM, Baqui AH, Zaman K (2007). Depressive symptoms among rural Bangladeshi mothers: implications for infant development. J Child Psychol Psychiatry.

[24] Duflo E, Glennerster R, Kremer M, Schultz TP, Strauss JA (2007). Using randomization in development economics research: a toolkit. Handbook of Development Economics.

[25] Coates J, Swindale A, Bilinsky P (2007). Household Food Insecurity Access Scale (HFIAS) for Measurement of Food Access: Indicator Guide. http://www.fao.org/fileadmin/user_upload/eufao-fsi4dm/doc-training/hfias.pdf.

[26] National Forum on Early Childhood Policy and Programs, National Scientific Council on The Developing Child (2011). Building the brain's “air traffic control” system: how early experiences shape the development of executive function: working paper no. 11. http://www.developingchild.harvard.edu/.

[27] Prado EL, Abbeddou S, Yakes Jimenez E (2017). Effects of an intervention on infant growth and development: evidence for different mechanisms at work. Matern Child Nutr.

[28] Ngure FM, Reid BM, Humphrey JH, Mbuya MN, Pelto G, Stoltzfus RJ (2014). Water, sanitation, and hygiene (WASH), environmental enteropathy, nutrition, and early child development: making the links. Ann NY Acad Sci.

[29] Bowen A, Agboatwalla M, Ayers T, Tobery T, Tariq M, Luby SP (2013). Sustained improvements in handwashing indicators more than 5 years after a cluster-randomised, community-based trial of handwashing promotion in Karachi, Pakistan. Trop Med Int Health.

[30] Neece CL, Green SA, Baker B (2012). Parenting stress and child behavioral problems: a transactional relationship across time. Am J Intellect Dev Disabil.

[31] Sudfeld CR, McCoy DC, Danaei G (2015). Linear growth and child development in low- and middle-income countries: a meta-analysis. Pediatrics.

[32] Prado EL, Abbeddou S, Adu-Afarwuah S (2016). Linear growth and child development in Burkina Faso, Ghana, and Malawi. Pediatrics.

[33] Prado EL, Adu-Afarwuah S, Lartey A (2016). Effects of pre- and post-natal lipid-based nutrient supplements on infant development in a randomized trial in Ghana. Early Hum Devel.

[34] Prado EL, Maleta K, Ashorn P (2016). Effects of maternal and child lipid-based nutrient supplements on infant development: a randomized trial in Malawi. Am J Clin Nutr.

[35] Prado EL, Dewey KG (2014). Nutrition and brain development in early life. Nutr Rev.

[36] Guerrant DI, Moore SR, Lima AA, Patrick PD, Schorling JB, Guerrant RL (1999). Association of early childhood diarrhea and cryptosporidiosis with impaired physical fitness and cognitive function four-seven years later in a poor urban community in northeast Brazil. Am J Trop Med Hyg.

[37] Jiang NM, Tofail F, Ma JZ (2017). Early life inflammation and neurodevelopmental outcome in Bangladeshi infants growing up in adversity. Am J Trop Med Hyg.

[38] Gillespie S, Menon P, Kennedy AL (2015). Scaling up impact on nutrition: what will it take?. Adv Nutr.

