# Supplementary material for: Effect of water quality, sanitation, hand washing, and nutritional interventions on child development in rural Bangladesh (WASH Benefits Bangladesh): a cluster-randomised controlled trial
Source: Lancet Child Adolesc Health. 2018 Apr;2(4):255–68. doi: 10.1016/S2352-4642(18)30031-2 (PMC5859216; doi:10.1016/S2352-4642(18)30031-2)
Supplement: Supplementary appendix [file mmc1.pdf]

THE LANCET
Child & Adolescent Health

Supplementary appendix

This appendix formed part of the original submission and has been peer reviewed. We post it as supplied by the authors.

Supplement to: Tofail F, Fernald LCH, Das KK, et al. Effect of water quality, sanitation, hand washing, and nutritional interventions on child development in rural Bangladesh (WASH Benefits Bangladesh): a cluster-randomised controlled trial. *Lancet Child Adolesc Health* 2018; published online Feb 12. http://dx.doi.org/10.1016/S2352-4642(18)30031-2.

Supplementary Table 1: Enrollment characteristics by compounds included in the analyses and lost to follow-up

	Included	Lost to follow
No. of compounds	4617	934
**Maternal**		
Age in year	23.9 (5.2)	23.1 (5.3)
Years of education	5.9 (3.4)	5.5 (3.5)
**Paternal**		
Years of education	4.9 (4.0)	4.6 (4.1)
Works in agriculture	1460 (32%)	286 (31%)
**Household**		
Number of persons	4.7 (2.2)	4.6 (2.3)
Has electricity	2771 (60%)	495 (53%)
Has a cement floor	494 (11%)	89 (10%)
Acres of agricultural land owned	0.15 (0.26)	0.14 (0.19)
**Drinking Water**		
Tubewell primary water source	3450 (75%)	658 (70%)
Stored water observed at home	2166 (47%)	477 (51%)
**Sanitation**		
Daily defecation in the open		
Adult men	327 (7%)	88 (10%)
Adult women	193 (4%)	43 (5%)
Children: 8-<15 years	192 (10%)	38 (10%)
Children: 3-<8 years	912 (37%)	170 (41%)
Children: 0-<3 years	808 (83%)	160 (85%)
Latrine		
Owned	2479 (54%)	497 (53%)
Concrete slab	4166 (94%)	813 (92%)
Functional water seal	1175 (30%)	195 (26%)
Visible stool on slab or floor	2154 (50%)	406 (47%)
Owned a potty	195 (4%)	49 (5%)
Human feces observed in the		
House	391 (8%)	69 (7%)
Child's play area	53 (1%)	10 (1%)
**Handwashing**		
Within 6 steps of latrine		
Has water	522 (12%)	84 (10%)
Has soap	282 (7%)	50 (6%)
Within 6 steps of kitchen		
Has water	358 (8%)	87 (10%)
Has soap	113 (3%)	19 (2%)

Data are n (%) or mean (SD). Percentages were estimated from slightly smaller denominators than those shown at the top of the table for the following variables due to missing values: father works in agriculture, open defecation, latrine has a concrete slab, latrine has a functional water seal, visible stool on latrine slab or floor, ownership of child potty, observed feces in the house or child's play area, handwashing variables.

Supplementary Table 2: Measures of intervention adherence by study arm at baseline and 1 and 2 years after delivery of interventions

	Control		Water (W)		Sanitation (S)		Handwashing (H)		WSH		Nutrition		Nutrition + WSH	
	N	n (%)	N	n (%)	N	n (%)	N	n (%)	N	n (%)	N	n (%)	N	n (%)
**Stored drinking water**														
Baseline	1382	666 (48)	698	353 (51)	696	341 (49)	688	347 (50)	702	304 (43)	699	301 (43)	686	331 (48)
Year 1	1151	503 (44)	611	587 (96)	583	245 (42)	585	266 (45)	605	588 (97)	581	229 (39)	600	577 (96)
Year 2	1138	485 (43)	598	567 (95)	585	260 (44)	570	267 (47)	588	558 (95)	574	225 (39)	586	569 (97)
**Stored drinking water has detectable free chlorine**														
[illegible]	[illegible]	–	0	–	0	–	0	–	0	–	0	–	0	–
Year 1	1150	0 (0)	601	467 (78)	583	0 (0)	585	1 (0)	592	467 (79)	579	0 (0)	588	472 (80)
Year 2	1138	0 (0)	581	488 (84)	585	0 (0)	570	0 (0)	579	471 (81)	574	0 (0)	578	501 (87)
**Latrine with a functional water seal**														
Baseline	1171	358 (31)	598	183 (31)	585	177 (30)	575	162 (28)	582	152 (26)	587	183 (31)	573	155 (27)
Year 1	1048	308 (29)	558	151 (27)	581	554 (95)	540	144 (27)	603	573 (95)	531	149 (28)	599	564 (94)
Year 2	1053	324 (31)	559	184 (33)	584	568 (97)	523	165 (32)	587	567 (97)	526	163 (31)	583	561 (96)
**No visible feces on latrine slab**														
Baseline	1307	625 (48)	666	350 (53)	642	332 (52)	642	335 (52)	653	289 (44)	649	331 (51)	643	298 (46)
Year 1	1089	658 (60)	587	358 (61)	581	516 (89)	562	324 (58)	604	522 (86)	554	333 (60)	599	527 (88)
Year 2	1085	612 (56)	579	338 (58)	585	502 (86)	543	324 (60)	587	484 (82)	541	313 (58)	583	495 (85)
**Handwashing location has water and soap**														
Baseline	1256	291 (23)	630	151 (24)	631	155 (25)	622	133 (21)	646	155 (24)	644	151 (23)	640	149 (23)
Year 1	1004	282 (28)	549	161 (29)	521	156 (30)	584	518 (89)	604	544 (90)	511	171 (33)	600	528 (88)
Year 2	1138	319 (28)	598	176 (29)	585	178 (30)	570	520 (91)	588	524 (89)	574	194 (34)	586	533 (91)
**LNS sachets consumed (% of expected†)**														
[illegible]	[illegible]	–	0	–	0	–	0	–	0	–	0	–	0	–
Year 1	0	–	0	–	0	–	0	–	0	–	531	493 (93)	543	513 (94)
Year 2	0		0	–	0	–	0	–	0	–	446	418 (94)	459	427 (93)

† Expected consumption was defined as 2 sachets per day.

Supplemetary Table 3: Effect of the intervention on motor miliestone attainment after 1 year of intervention - adjusted† analyses

Outcome, Arm	N	Hazard Ratio vs. Control (95% CI)	Hazard Ratio vs. WSH (95% CI)	Hazard Ratio vs. Nutrition (95% CI)
**Standing with assistance**				
Control	1161	Ref		
Water	598	1.01 (0.87, 1.17)		
Sanitation	593	1.04 (0.89, 1.21)		
Handwashing	584	0.93 (0.81, 1.07)		
WSH	594	0.94 (0.82, 1.09)	Ref	
Nutrition	580	1.04 (1.04, 1.04)		Ref
Nutrition+WSH	603	1.02 (1.02, 1.02)	1.04 (1.04, 1.04)	0.94 (0.79, 1.11)
**Hands and knees crawling**				
Control	1171	Ref		
Water	601	0.96 (0.84, 1.09)		
Sanitation	593	0.98 (0.98, 0.98)		
Handwashing	589	0.84 (0.74, 0.96)		
WSH	595	0.94 (0.94, 0.94)	Ref	
Nutrition	584	1.00 (0.88, 1.13)		Ref
Nutrition+WSH	606	0.96 (0.84, 1.09)	1.07 (0.92, 1.25)	0.98 (0.98, 0.98)
**Walking with assistance**				
Control	1160	Ref		
Water	598	1.03 (1.03, 1.03)		
Sanitation	591	1.10 (0.95, 1.27)		
Handwashing	583	0.86 (0.74, 0.99)		
WSH	594	0.99 (0.99, 0.99)	Ref	
Nutrition	580	1.13 (0.97, 1.31)		Ref
Nutrition+WSH	604	1.01 (1.01, 1.01)	1.02 (1.02, 1.02)	0.96 (0.96, 0.96)
**Standing alone**				
Control	1165	Ref		
Water	600	1.17 (0.99, 1.38)		
Sanitation	591	1.07 (0.90, 1.27)		
Handwashing	585	0.96 (0.96, 0.96)		
WSH	592	1.01 (0.84, 1.20)	Ref	
Nutrition	582	1.04 (1.04, 1.04)		Ref
Nutrition+WSH	607	1.16 (0.98, 1.38)	1.20 (0.99, 1.47)	1.04 (1.04, 1.04)
**Walking alone**				
Control	1165	Ref		
Water	600	1.27 (1.04, 1.57)		
Sanitation	592	1.03 (1.03, 1.03)		
Handwashing	585	1.02 (1.02, 1.02)		
WSH	593	0.98 (0.98, 0.98)	Ref	
Nutrition	581	1.30 (1.05, 1.61)		Ref
Nutrition+WSH	607	1.23 (1.00, 1.52)	1.34 (1.03, 1.74)	0.98 (0.98, 0.98)

† adjusted for child age, child sex, maternal age, maternal height in cm, parents' education, number of children <18 years in household, total number of people in the compound, food insequrity of household measured using Household Hunger Scale, housing materials, household assets, distance to water source, field staff team member who admistered motor milestone attainment questionnaire, and month of measurement.

Supplementary Table 4: Effect of the intervention on the communication, gross motor, and personal-scocial scales of the Extended Ages and Stages Questionnaire after 2 years of intervention- adjusted analyses

Outcome, Arm	N	Mean (SD)	Adjusted Difference Model 1 †	Adjusted Difference Model 2‡
**Communication z score**				
Control	1099	0.00 (1.00)	Ref	Ref
Water	565	0.09 (0.96)	0.10 (-0.02, 0.21)	0.04 (-0.05, 0.13)
Sanitation	555	0.20 (0.95)	0.21 (0.09, 0.33)	0.16 (0.06, 0.25)
Handwashing	543	0.20 (0.94)	0.21 (0.10, 0.31)	0.13 (0.04, 0.22)
WSH	547	0.15 (0.93)	0.14 (0.03, 0.26)	0.14 (0.05, 0.23)
Nutrition	541	0.19 (0.86)	0.20 (0.10, 0.29)	0.16 (0.08, 0.24)
Nutrition+WSH	553	0.25 (0.90)	0.26 (0.16, 0.36)	0.17 (0.09, 0.26)
**Gross Motor z score**				
Control	1099	0.00 (1.00)	Ref	Ref
Water	557	0.01 (0.93)	0.01 (-0.11, 0.13)	-0.02 (-0.13, 0.09)
Sanitation	549	0.12 (0.93)	0.13 (0.00, 0.25)	0.09 (-0.03, 0.21)
Handwashing	535	0.10 (0.93)	0.12 (0.00, 0.23)	0.10 (-0.01, 0.21)
WSH	539	0.16 (0.87)	0.16 (0.05, 0.27)	0.15 (0.05, 0.26)
Nutrition	528	0.18 (0.95)	0.19 (0.08, 0.30)	0.19 (0.08, 0.30)
Nutrition+WSH	546	0.14 (0.94)	0.14 (0.03, 0.25)	0.12 (0.01, 0.23)
**Personal-social z score**				
Control	1099	0.00 (1.00)	Ref	Ref
Water	557	0.11 (0.91)	0.13 (0.01, 0.25)	0.10 (-0.01, 0.22)
Sanitation	544	0.28 (0.97)	0.29 (0.18, 0.40)	0.26 (0.15, 0.37)
Handwashing	528	0.26 (0.94)	0.28 (0.17, 0.40)	0.26 (0.16, 0.37)
WSH	538	0.28 (1.01)	0.27 (0.16, 0.38)	0.27 (0.18, 0.37)
Nutrition	528	0.22 (0.97)	0.22 (0.11, 0.33)	0.20 (0.11, 0.30)
Nutrition+WSH	538	0.34 (0.98)	0.35 (0.24, 0.46)	0.33 (0.22, 0.43)
**Combined z score**				
Control	1099	0.00 (1.00)	Ref	Ref
Water	539	0.14 (0.86)	0.15 (0.04, 0.26)	0.10 (0.01, 0.19)
Sanitation	531	0.31 (0.86)	0.31 (0.19, 0.43)	0.26 (0.15, 0.36)
Handwashing	502	0.27 (0.87)	0.29 (0.19, 0.39)	0.22 (0.13, 0.32)
WSH	519	0.25 (0.90)	0.24 (0.14, 0.35)	0.23 (0.13, 0.33)
Nutrition	506	0.27 (0.83)	0.28 (0.18, 0.37)	0.25 (0.17, 0.34)
Nutrition+WSH	522	0.36 (0.81)	0.37 (0.27, 0.46)	0.30 (0.22, 0.39)

† Model 1: adjusted for child age in days only;

‡ Model 2: adjusted for covariate used to adjust in model 1, child sex, maternal age, maternal height in cm, parents' education, number of children <18 years in household, total number of people in the compound, food insequrity of household measured using Household Hunger Scale, housing materials, household assets, distance to water source, field staff team member who admistered Extended Ages and Stages Questionnaire, and month of measurement.

Supplementary Table 5: Effect of the intervention on Communication Development Inventory after 1 year of intervention

Outcome, Arm	N	Mean (SD)	Mean Difference vs. Control (95% CI)	Mean Difference vs. WSH (95% CI)	Mean Difference vs. Nutrition (95% CI)
**CDI†- understand z-score**					
Control	1172	0.00 (1.00)	Ref		
Water	570	0.10 (0.99)	0.12 (0.02, 0.21)		
Sanitation	556	0.02 (0.93)	0.02 (-0.08, 0.13)		
Handwashing	562	0.10 (0.95)	0.11 (0.01, 0.21)		
WSH	572	0.10 (0.96)	0.10 (-0.02, 0.22)	Ref	
Nutrition	551	0.13 (0.94)	0.14 (0.03, 0.25)		Ref
Nutrition+WSH	584	0.11 (0.92)	0.13 (0.02, 0.23)	0.00 (-0.14, 0.15)	-0.02 (-0.15, 0.12)
**CDI†- say z-score**					
Control	1172	0.00 (1.00)	Ref		
Water	594	0.03 (0.90)	0.03 (-0.06, 0.13)		
Sanitation	579	0.04 (0.99)	0.04 (-0.06, 0.15)		
Handwashing	577	0.10 (0.92)	0.10 (-0.01, 0.22)		
WSH	582	0.02 (0.89)	0.01 (-0.09, 0.12)	Ref	
Nutrition	574	0.10 (0.90)	0.11 (-0.01, 0.23)		Ref
Nutrition+WSH	598	0.06 (0.88)	0.07 (-0.04, 0.18)	0.03 (-0.09, 0.16)	-0.04 (-0.19, 0.11)

† The MacArthur-Bates Communicative Development Inventories.

Supplementary Table 6: Effect of intervention on maternal depressive symptoms and HOME scale after 1 and 2 year of intervention

		Mean (SD)	Mean Difference vs. Control (CI)
**Maternal depressive symptoms after 1 year**			
Control	1172	0.00 (1.00)	Ref
Water	526	-0.19 (0.73)	-0.19 (-0.28, -0.09)
Sanitation	521	-0.20 (0.69)	-0.20 (-0.28, -0.13)
Handwashing	509	-0.22 (0.69)	-0.22 (-0.30, -0.13)
WSH	512	-0.17 (0.78)	-0.17 (-0.26, -0.08)
Nutrition	510	-0.31 (0.71)	-0.31 (-0.40, -0.22)
Nutrition+WSH	532	-0.27 (0.72)	-0.28 (-0.36, -0.19)
**Maternal depressive symptoms after 2 year**			
Control	1106	0.00 (1.00)	Ref
Water	515	-0.22 (0.73)	-0.23 (-0.33, -0.13)
Sanitation	506	-0.23 (0.74)	-0.23 (-0.32, -0.15)
Handwashing	484	-0.18 (0.77)	-0.19 (-0.28, -0.10)
WSH	512	-0.19 (0.78)	-0.19 (-0.30, -0.09)
Nutrition	501	-0.24 (0.78)	-0.24 (-0.34, -0.14)
Nutrition+WSH	508	-0.27 (0.78)	-0.29 (-0.38, -0.19)
**HOME scale after 1 year**			
Control	1172	0.00 (1.00)	Ref
Water	588	0.12 (0.96)	0.11 (-0.02, 0.23)
Sanitation	583	0.18 (0.96)	0.16 (0.05, 0.28)
Handwashing	575	0.18 (0.99)	0.18 (0.03, 0.33)
WSH	582	0.09 (0.91)	0.08 (-0.05, 0.21)
Nutrition	566	0.09 (0.95)	0.09 (-0.04, 0.22)
Nutrition+WSH	597	0.16 (0.99)	0.16 (0.02, 0.31)
**HOME scale after 2 year**			
Control	1106	0.00 (1.00)	Ref
Water	564	0.13 (1.01)	0.13 (0.01, 0.25)
Sanitation	551	0.10 (0.96)	0.11 (-0.03, 0.24)
Handwashing	536	0.12 (0.93)	0.12 (-0.01, 0.25)
WSH	547	0.04 (0.88)	0.03 (-0.08, 0.14)
Nutrition	529	0.19 (0.97)	0.20 (0.09, 0.31)
Nutrition+WSH	545	0.23 (0.97)	0.24 (0.10, 0.37)

Supplementary Table 7: Effect modification with child gender

	Male			Female			
**Outcome**	**N**	**Mean (SD)**	**Mean Difference vs. Control (95% CI)**	**N**	**Mean (SD)**	**Mean Difference vs. Control (95% CI)**	**P for Interaction**
**Communication z-score**							
Control	555	-0.06 (0.99)		544	0.06 (1.00)		
Water	279	0.10 (1.00)	0.15 (0.00, 0.31)	286	0.09 (0.92)	0.04 (-0.12, 0.19)	0.28
Sanitation	280	0.09 (1.03)	0.13 (-0.03, 0.30)	275	0.32 (0.84)	0.29 (0.14, 0.44)	0.13
Handwashing	263	0.17 (0.96)	0.26 (0.10, 0.41)	280	0.22 (0.92)	0.15 (-0.01, 0.31)	0.39
WSH	282	0.11 (0.98)	0.16 (0.00, 0.32)	265	0.20 (0.88)	0.12 (-0.01, 0.26)	0.69
Nutrition	276	0.14 (0.90)	0.21 (0.09, 0.34)	265	0.25 (0.81)	0.17 (0.03, 0.31)	0.64
Nutrition+WSH	263	0.15 (0.99)	0.21 (0.06, 0.36)	290	0.35 (0.81)	0.30 (0.17, 0.43)	0.36
N+WSHvsWSH			0.07 (-0.12, 0.25)			0.15 (-0.01, 0.31)	0.47
N+WSH vs N			0.01 (-0.18, 0.19)			0.12 (-0.03, 0.28)	0.33
**Gross Motor z-score**							
Control	555	0.11 (0.99)		544	-0.11 (1.00)		
Water	274	0.13 (0.93)	0.03 (-0.14, 0.19)	283	-0.11 (0.92)	0.00 (-0.15, 0.15)	0.80
Sanitation	277	0.23 (0.95)	0.13 (-0.04, 0.30)	272	0.02 (0.89)	0.12 (-0.02, 0.26)	0.92
Handwashing	259	0.19 (0.93)	0.12 (-0.04, 0.28)	276	0.02 (0.93)	0.12 (-0.04, 0.27)	0.98
WSH	281	0.21 (0.88)	0.12 (-0.01, 0.26)	258	0.11 (0.85)	0.19 (0.03, 0.34)	0.51
Nutrition	269	0.28 (0.90)	0.22 (0.08, 0.35)	259	0.07 (0.99)	0.16 (0.00, 0.32)	0.60
Nutrition+WSH	259	0.16 (1.06)	0.07 (-0.10, 0.25)	287	0.12 (0.82)	0.22 (0.07, 0.36)	0.21
N+WSH vs WSH			-0.05 (-0.22, 0.12)			0.04 (-0.11, 0.19)	0.43
N+WSH vs N			-0.11 (-0.30, 0.08)			0.03 (-0.16, 0.21)	0.27
**Personal-social z-score**							
Control	555	-0.18 (0.97)		544	0.18 (1.00)		
Water	274	-0.04 (0.94)	0.15 (-0.01, 0.30)	283	0.26 (0.85)	0.10 (-0.06, 0.25)	0.62
Sanitation	272	0.07 (0.95)	0.25 (0.10, 0.40)	272	0.49 (0.95)	0.33 (0.16, 0.49)	0.50
Handwashing	255	0.11 (0.97)	0.32 (0.16, 0.48)	273	0.40 (0.89)	0.23 (0.08, 0.38)	0.41
WSH	279	0.11 (1.04)	0.29 (0.13, 0.44)	259	0.45 (0.94)	0.26 (0.11, 0.41)	0.81
Nutrition	272	0.07 (1.01)	0.29 (0.13, 0.44)	256	0.37 (0.90)	0.15 (0.01, 0.30)	0.21
Nutrition+WSH	250	0.20 (0.97)	0.38 (0.23, 0.53)	288	0.45 (0.98)	0.30 (0.14, 0.46)	0.46
N+WSH vs WSH			0.10 (-0.07, 0.28)			0.01 (-0.17, 0.18)	0.43
N+WSH vs N			0.15 (-0.04, 0.34)			0.09 (-0.08, 0.27)	0.64
**Combined z-score**							
Control	555	-0.06 (0.98)		544	0.06 (1.02)		
Water	262	0.13 (0.88)	0.20 (0.06, 0.34)	277	0.15 (0.84)	0.10 (-0.06, 0.25)	0.31
Sanitation	266	0.23 (0.89)	0.28 (0.13, 0.42)	265	0.39 (0.82)	0.34 (0.19, 0.50)	0.48
Handwashing	241	0.24 (0.90)	0.34 (0.19, 0.49)	261	0.30 (0.83)	0.23 (0.09, 0.38)	0.33
WSH	264	0.21 (0.94)	0.28 (0.13, 0.42)	255	0.29 (0.86)	0.21 (0.06, 0.37)	0.54
Nutrition	258	0.22 (0.86)	0.31 (0.17, 0.45)	248	0.33 (0.80)	0.25 (0.10, 0.39)	0.58
Nutrition+WSH	240	0.29 (0.83)	0.35 (0.21, 0.50)	282	0.42 (0.79)	0.37 (0.23, 0.51)	0.86
N+WSH vs WSH			0.08 (-0.09, 0.26)			0.14 (-0.03, 0.31)	0.65
N+WSH vs N			0.08 (-0.09, 0.25)			0.10 (-0.06, 0.25)	0.87

Supplementary Table 8: Effect modification with maternal parity

	Multiparous			Nulliparous			
Outcome	N	Mean (SD)	Mean Difference vs. Control (95% CI)	N	Mean (SD)	Mean Difference vs. Control (95% CI)	P for Interaction
**Communication z-score**							
Control	617	-0.07 (1.02)		262	0.18 (0.90)		
Water	349	0.03 (0.96)	0.13 (-0.03, 0.29)	165	0.20 (0.97)	0.00 (-0.20, 0.20)	0.31
Sanitation	362	0.15 (0.97)	0.21 (0.06, 0.36)	154	0.34 (0.94)	0.22 (0.02, 0.41)	0.93
Handwashing	351	0.13 (0.99)	0.20 (0.05, 0.35)	153	0.31 (0.88)	0.19 (-0.02, 0.40)	0.95
WSH	340	0.15 (0.89)	0.22 (0.09, 0.36)	142	0.21 (0.88)	0.00 (-0.19, 0.20)	0.059
Nutrition	302	0.17 (0.87)	0.24 (0.11, 0.37)	168	0.25 (0.83)	0.08 (-0.09, 0.25)	0.16
Nutrition+WSH	327	0.24 (0.93)	0.32 (0.18, 0.46)	163	0.36 (0.83)	0.22 (0.05, 0.39)	0.40
N+WSH vs WSH			0.10 (-0.08, 0.28)			0.17 (-0.05, 0.40)	0.62
N+WSH vs N			0.05 (-0.11, 0.21)			0.15 (-0.05, 0.35)	0.46
**Gross Motor z-score**							
Control	617	0.01 (0.95)		262	0.00 (0.97)		
Water	346	0.01 (0.83)	0.00 (-0.13, 0.13)	161	0.02 (1.14)	0.01 (-0.21, 0.22)	0.95
Sanitation	362	0.08 (0.95)	0.05 (-0.10, 0.19)	151	0.23 (0.90)	0.26 (0.07, 0.45)	0.071
Handwashing	347	0.08 (0.94)	0.08 (-0.07, 0.22)	150	0.14 (0.91)	0.14 (-0.05, 0.33)	0.57
WSH	336	0.12 (0.82)	0.10 (-0.04, 0.24)	138	0.19 (0.88)	0.20 (-0.02, 0.43)	0.44
Nutrition	295	0.20 (0.89)	0.19 (0.04, 0.33)	162	0.20 (1.01)	0.20 (0.01, 0.40)	0.88
Nutrition+WSH	328	0.16 (0.91)	0.15 (0.00, 0.29)	158	0.13 (0.96)	0.14 (-0.05, 0.33)	0.98
N+WSH vs WSH			0.04 (-0.11, 0.18)			-0.04 (-0.28, 0.20)	0.61
N+WSH vs N			-0.05 (-0.24, 0.13)			-0.05 (-0.33, 0.22)	0.99
**Personal-social z-score**							
Control	617	-0.03 (1.01)		262	0.09 (0.95)		
Water	346	0.07 (0.87)	0.14 (0.00, 0.28)	163	0.24 (0.99)	0.17 (-0.02, 0.36)	0.74
Sanitation	356	0.25 (0.99)	0.29 (0.15, 0.44)	151	0.37 (0.91)	0.32 (0.11, 0.53)	0.81
Handwashing	343	0.25 (0.94)	0.29 (0.15, 0.44)	148	0.33 (0.96)	0.31 (0.10, 0.52)	0.92
WSH	339	0.30 (0.99)	0.36 (0.22, 0.50)	139	0.28 (1.02)	0.17 (-0.05, 0.40)	0.16
Nutrition	296	0.21 (0.98)	0.24 (0.09, 0.38)	163	0.23 (0.93)	0.17 (-0.02, 0.37)	0.61
Nutrition+WSH	318	0.24 (0.96)	0.30 (0.15, 0.44)	158	0.53 (0.96)	0.49 (0.27, 0.70)	0.14
N+WSH vs WSH			-0.05 (-0.22, 0.12)			0.26 (0.00, 0.52)	0.063
N+WSH vs N			0.04 (-0.15, 0.22)			0.28 (0.05, 0.52)	0.12
**Combined z-score**							
Control	617	-0.04 (1.00)		262	0.13 (0.94)		
Water	330	0.09 (0.81)	0.17 (0.04, 0.31)	160	0.25 (0.95)	0.11 (-0.07, 0.29)	0.55
Sanitation	344	0.26 (0.88)	0.30 (0.15, 0.45)	149	0.44 (0.81)	0.34 (0.14, 0.53)	0.73
Handwashing	323	0.25 (0.89)	0.30 (0.16, 0.45)	143	0.33 (0.85)	0.25 (0.06, 0.44)	0.66
WSH	325	0.28 (0.82)	0.33 (0.20, 0.46)	134	0.24 (0.97)	0.10 (-0.11, 0.31)	0.074
Nutrition	282	0.28 (0.84)	0.33 (0.19, 0.46)	158	0.31 (0.79)	0.20 (0.03, 0.38)	0.29
Nutrition+WSH	313	0.31 (0.82)	0.37 (0.25, 0.50)	149	0.51 (0.68)	0.41 (0.26, 0.57)	0.67
N+WSH vs WSH			0.05 (-0.10, 0.20)			0.27 (0.05, 0.49)	0.11
N+WSH vs N			0.02 (-0.14, 0.18)			0.20 (0.00, 0.40)	0.19

Supplementary Table 9: Effect modification with maternal age

Outcome	Mother over 20y old N	Mean (SD)	Mean Difference vs. Control (95% CI)	Mother 20y old or younger N	Mean (SD)	Mean Difference vs. Control (95% CI)	P for Interaction
**Communication z-score**							
Control	735	-0.05 (1.03)		360	0.09 (0.94)		
Water	368	0.06 (0.97)	0.11 (-0.03, 0.26)	195	0.15 (0.92)	0.06 (-0.13, 0.24)	0.63
Sanitation	375	0.20 (0.94)	0.23 (0.10, 0.37)	180	0.21 (0.95)	0.17 (-0.01, 0.36)	0.57
Handwashing	347	0.17 (0.97)	0.22 (0.09, 0.35)	194	0.25 (0.88)	0.20 (0.04, 0.36)	0.83
WSH	377	0.20 (0.91)	0.24 (0.11, 0.37)	163	0.06 (0.98)	-0.04 (-0.22, 0.14)	0.0080
Nutrition	356	0.21 (0.85)	0.24 (0.14, 0.35)	182	0.17 (0.87)	0.10 (-0.09, 0.28)	0.18
Nutrition+WSH	357	0.22 (0.95)	0.27 (0.13, 0.40)	194	0.31 (0.81)	0.24 (0.09, 0.39)	0.81
N+WSH vs WSH			0.05 (-0.13, 0.22)			0.25 (0.04, 0.45)	0.15
N+WSH vs N			0.00 (-0.15, 0.15)			0.20 (0.00, 0.39)	0.11
**Gross Motor z-score**							
Control	735	0.01 (1.04)		360	-0.03 (0.92)		
Water	364	0.00 (0.92)	-0.01 (-0.15, 0.12)	191	0.03 (0.95)	0.06 (-0.12, 0.23)	0.49
Sanitation	377	0.13 (0.87)	0.10 (-0.04, 0.24)	172	0.10 (1.03)	0.18 (0.00, 0.35)	0.44
Handwashing	339	0.09 (0.99)	0.09 (-0.06, 0.24)	194	0.12 (0.82)	0.17 (0.02, 0.32)	0.45
WSH	378	0.20 (0.83)	0.17 (0.05, 0.30)	155	0.05 (0.95)	0.09 (-0.12, 0.30)	0.50
Nutrition	351	0.18 (0.94)	0.17 (0.03, 0.32)	174	0.17 (0.96)	0.21 (0.05, 0.37)	0.73
Nutrition+WSH	356	0.14 (0.94)	0.14 (0.00, 0.27)	188	0.12 (0.94)	0.16 (-0.02, 0.34)	0.83
N+WSH vs WSH			-0.04 (-0.19, 0.11)			0.09 (-0.11, 0.29)	0.34
N+WSH vs N			-0.05 (-0.20, 0.10)			-0.03 (-0.29, 0.22)	0.92
**Personal-social z-score**							
Control	735	-0.02 (1.02)		360	0.03 (0.95)		
Water	364	0.08 (0.89)	0.12 (-0.01, 0.25)	191	0.16 (0.95)	0.13 (-0.07, 0.32)	0.94
Sanitation	369	0.28 (1.00)	0.30 (0.16, 0.44)	175	0.28 (0.92)	0.28 (0.08, 0.48)	0.89
Handwashing	334	0.25 (0.94)	0.28 (0.14, 0.42)	192	0.29 (0.93)	0.30 (0.13, 0.47)	0.85
WSH	377	0.32 (1.02)	0.34 (0.19, 0.48)	154	0.19 (0.98)	0.13 (-0.05, 0.31)	0.088
Nutrition	347	0.19 (0.99)	0.21 (0.08, 0.33)	178	0.25 (0.93)	0.23 (0.06, 0.40)	0.79
Nutrition+WSH	348	0.25 (1.00)	0.28 (0.14, 0.41)	188	0.50 (0.93)	0.48 (0.31, 0.65)	0.056
N+WSH vs WSH			-0.04 (-0.22, 0.14)			0.28 (0.05, 0.50)	0.044
N+WSH vs N			0.06 (-0.12, 0.25)			0.27 (0.06, 0.49)	0.15
**Combined z-score**							
Control	735	-0.03 (1.03)		360	0.05 (0.93)		
Water	349	0.11 (0.86)	0.15 (0.02, 0.28)	188	0.20 (0.86)	0.14 (-0.03, 0.31)	0.90
Sanitation	359	0.30 (0.87)	0.31 (0.17, 0.45)	172	0.33 (0.83)	0.32 (0.14, 0.50)	0.92
Handwashing	315	0.27 (0.85)	0.30 (0.17, 0.43)	186	0.28 (0.89)	0.27 (0.11, 0.43)	0.77
WSH	363	0.31 (0.86)	0.33 (0.19, 0.46)	150	0.12 (0.98)	0.07 (-0.12, 0.25)	0.021
Nutrition	334	0.27 (0.85)	0.30 (0.18, 0.41)	169	0.26 (0.79)	0.24 (0.07, 0.40)	0.54
Nutrition+WSH	341	0.30 (0.87)	0.34 (0.21, 0.46)	179	0.45 (0.69)	0.42 (0.27, 0.57)	0.41
N+WSH vs WSH			0.02 (-0.15, 0.19)			0.32 (0.12, 0.53)	0.030
N+WSH vs N			0.03 (-0.12, 0.18)			0.22 (0.05, 0.40)	0.083

Supplementary Table 10: Effect modification with maternal education

	Low Education			High Education			
Outcome	N	Mean (SD)	Mean Difference vs. Control (95% CI)	N	Mean (SD)	Mean Difference vs. Control (95% CI)	P for Interaction
**Communication z-score**							
Control	971	-0.04 (1.01)		128	0.31 (0.89)		
Water	499	0.06 (0.96)	0.10 (-0.02, 0.21)	66	0.36 (0.92)	0.10 (-0.19, 0.40)	0.95
Sanitation	491	0.17 (0.96)	0.21 (0.09, 0.33)	64	0.45 (0.78)	0.20 (-0.06, 0.47)	0.95
Handwashing	482	0.14 (0.96)	0.19 (0.07, 0.32)	61	0.64 (0.59)	0.36 (0.11, 0.60)	0.27
WSH	471	0.12 (0.95)	0.14 (0.02, 0.26)	76	0.34 (0.82)	0.11 (-0.17, 0.39)	0.83
Nutrition	482	0.17 (0.88)	0.21 (0.11, 0.31)	59	0.42 (0.59)	0.09 (-0.12, 0.31)	0.35
Nutrition+WSH	488	0.20 (0.91)	0.25 (0.15, 0.35)	65	0.63 (0.78)	0.34 (0.08, 0.60)	0.53
N+WSH vs WSH			0.10 (-0.05, 0.24)			0.28 (-0.01, 0.57)	0.27
N+WSH vs N			0.05 (-0.08, 0.18)			0.21 (-0.04, 0.46)	0.23
**Gross Motor z-score**							
Control	971	-0.01 (1.00)		128	0.09 (1.01)		
Water	494	0.01 (0.91)	0.02 (-0.09, 0.13)	63	0.02 (1.05)	-0.07 (-0.41, 0.27)	0.58
Sanitation	486	0.08 (0.93)	0.10 (-0.02, 0.22)	63	0.44 (0.85)	0.34 (0.01, 0.67)	0.14
Handwashing	475	0.09 (0.94)	0.11 (-0.02, 0.24)	60	0.23 (0.84)	0.18 (-0.12, 0.47)	0.68
WSH	468	0.15 (0.87)	0.15 (0.04, 0.26)	71	0.20 (0.85)	0.17 (-0.12, 0.46)	0.90
Nutrition	472	0.16 (0.95)	0.18 (0.07, 0.28)	56	0.39 (0.88)	0.30 (-0.05, 0.66)	0.48
Nutrition+WSH	482	0.12 (0.92)	0.12 (0.00, 0.24)	64	0.29 (1.07)	0.31 (0.00, 0.61)	0.26
N+WSH vs WSH			-0.04 (-0.15, 0.08)			0.20 (-0.20, 0.59)	0.27
N+WSH vs N			-0.05 (-0.21, 0.10)			0.00 (-0.37, 0.37)	0.77
**Personal-social z-score**							
Control	971	-0.02 (1.00)		128	0.16 (1.02)		
Water	493	0.09 (0.90)	0.13 (0.00, 0.25)	64	0.29 (0.95)	0.14 (-0.20, 0.48)	0.93
Sanitation	481	0.27 (0.97)	0.30 (0.18, 0.42)	63	0.37 (0.99)	0.23 (-0.07, 0.54)	0.69
Handwashing	469	0.25 (0.94)	0.29 (0.16, 0.42)	59	0.35 (0.90)	0.23 (-0.07, 0.54)	0.74
WSH	465	0.25 (1.02)	0.26 (0.14, 0.38)	73	0.41 (0.93)	0.30 (0.00, 0.61)	0.80
Nutrition	472	0.20 (0.97)	0.22 (0.11, 0.34)	56	0.37 (0.94)	0.19 (-0.09, 0.47)	0.84
Nutrition+WSH	478	0.30 (0.97)	0.33 (0.21, 0.45)	60	0.66 (1.03)	0.55 (0.22, 0.87)	0.20
N+WSH vs WSH			0.05 (-0.09, 0.19)			0.27 (-0.08, 0.63)	0.23
N+WSH vs N			0.10 (-0.05, 0.25)			0.41 (0.06, 0.75)	0.096
**Combined z-score**							
Control	971	-0.03 (1.00)		128	0.26 (0.94)		
Water	475	0.12 (0.84)	0.16 (0.05, 0.27)	64	0.30 (1.00)	0.07 (-0.25, 0.39)	0.57
Sanitation	471	0.28 (0.86)	0.32 (0.21, 0.44)	60	0.49 (0.85)	0.25 (-0.06, 0.55)	0.62
Handwashing	442	0.24 (0.87)	0.29 (0.18, 0.41)	60	0.51 (0.84)	0.27 (-0.02, 0.56)	0.87
WSH	451	0.22 (0.92)	0.23 (0.11, 0.34)	68	0.48 (0.71)	0.32 (0.06, 0.59)	0.52
Nutrition	454	0.25 (0.84)	0.29 (0.19, 0.39)	52	0.44 (0.69)	0.20 (-0.06, 0.46)	0.49
Nutrition+WSH	462	0.33 (0.79)	0.37 (0.27, 0.47)	60	0.59 (0.94)	0.38 (0.07, 0.69)	0.92
N+WSH vs WSH			0.12 (-0.01, 0.26)			0.13 (-0.19, 0.45)	0.98
N+WSH vs N			0.09 (-0.04, 0.21)			0.14 (-0.18, 0.45)	0.77

Supplementary Table 11: Effect modification with food insecurity

	Little to no hunger			Moderate to severe hunger			
**Outcome**	**N**	**Mean (SD)**	**Mean Difference vs. Control (95% CI)**	**N**	**Mean (SD)**	**Mean Difference vs. Control (95% CI)**	**P for Interaction**
**Communication z-score**							
Control	847	0.07 (0.96)		252	-0.24 (1.10)		
Water	449	0.13 (0.93)	0.07 (-0.05, 0.19)	116	-0.04 (1.03)	0.18 (-0.07, 0.43)	0.39
Sanitation	430	0.26 (0.92)	0.19 (0.07, 0.31)	125	0.00 (1.01)	0.26 (0.03, 0.49)	0.58
Handwashing	421	0.23 (0.93)	0.17 (0.06, 0.28)	122	0.07 (0.97)	0.33 (0.10, 0.57)	0.18
WSH	427	0.16 (0.93)	0.08 (-0.05, 0.20)	120	0.12 (0.94)	0.37 (0.15, 0.59)	0.016
Nutrition	426	0.26 (0.83)	0.19 (0.09, 0.28)	115	-0.06 (0.89)	0.19 (-0.02, 0.40)	0.99
Nutrition+WSH	448	0.29 (0.83)	0.22 (0.12, 0.32)	105	0.08 (1.16)	0.36 (0.09, 0.63)	0.35
N+WSH vs WSH			0.14 (0.00, 0.29)			-0.02 (-0.33, 0.29)	0.34
N+WSH vs N			0.04 (-0.09, 0.16)			0.17 (-0.14, 0.48)	0.43
**Gross Motor z-score**							
Control	847	0.06 (0.99)		252	-0.19 (1.02)		
Water	442	0.04 (0.89)	-0.01 (-0.14, 0.12)	115	-0.11 (1.05)	0.05 (-0.21, 0.31)	0.71
Sanitation	426	0.19 (0.92)	0.13 (0.00, 0.26)	123	-0.09 (0.91)	0.10 (-0.11, 0.31)	0.78
Handwashing	418	0.11 (0.97)	0.08 (-0.05, 0.20)	117	0.06 (0.79)	0.25 (0.03, 0.47)	0.13
WSH	417	0.15 (0.88)	0.09 (-0.04, 0.22)	122	0.20 (0.82)	0.38 (0.19, 0.58)	0.016
Nutrition	415	0.25 (0.94)	0.18 (0.07, 0.29)	113	-0.06 (0.94)	0.21 (-0.02, 0.45)	0.76
Nutrition+WSH	438	0.17 (0.90)	0.12 (0.00, 0.24)	108	0.02 (1.06)	0.20 (-0.06, 0.45)	0.59
N+WSH vs WSH			0.01 (-0.12, 0.14)			-0.09 (-0.35, 0.16)	0.48
N+WSH vs N			-0.08 (-0.23, 0.08)			0.06 (-0.27, 0.39)	0.46
**Personal-social z-score**							
Control	847	0.07 (0.98)		252	-0.24 (1.03)		
Water	440	0.14 (0.87)	0.08 (-0.05, 0.21)	117	0.03 (1.04)	0.27 (0.00, 0.54)	0.20
Sanitation	424	0.33 (0.99)	0.28 (0.14, 0.41)	120	0.09 (0.89)	0.32 (0.11, 0.53)	0.76
Handwashing	410	0.29 (0.93)	0.25 (0.13, 0.37)	118	0.16 (0.95)	0.39 (0.21, 0.57)	0.14
WSH	418	0.27 (1.00)	0.20 (0.07, 0.32)	120	0.30 (1.02)	0.53 (0.30, 0.75)	0.0090
Nutrition	417	0.27 (0.94)	0.20 (0.09, 0.30)	111	0.02 (1.03)	0.28 (0.03, 0.54)	0.50
Nutrition+WSH	436	0.38 (0.98)	0.30 (0.18, 0.43)	102	0.18 (1.01)	0.47 (0.25, 0.69)	0.20
N+WSH vs WSH			0.11 (-0.03, 0.25)			-0.10 (-0.40, 0.21)	0.21
N+WSH vs N			0.13 (-0.02, 0.27)			0.15 (-0.17, 0.46)	0.90
**Combined z-score**							
Control	847	0.08 (0.96)		252	-0.28 (1.09)		
Water	429	0.18 (0.83)	0.11 (0.00, 0.23)	110	-0.01 (0.95)	0.25 (0.02, 0.48)	0.29
Sanitation	413	0.37 (0.85)	0.29 (0.17, 0.42)	118	0.09 (0.85)	0.36 (0.15, 0.57)	0.55
Handwashing	392	0.30 (0.86)	0.24 (0.14, 0.34)	110	0.16 (0.87)	0.46 (0.26, 0.66)	0.028
WSH	404	0.26 (0.91)	0.17 (0.05, 0.30)	115	0.23 (0.86)	0.49 (0.28, 0.70)	0.0070
Nutrition	399	0.33 (0.81)	0.25 (0.16, 0.35)	107	0.04 (0.87)	0.34 (0.12, 0.56)	0.44
Nutrition+WSH	426	0.37 (0.82)	0.29 (0.18, 0.40)	96	0.29 (0.79)	0.59 (0.40, 0.79)	0.010
N+WSH vs WSH			0.12 (-0.02, 0.26)			0.09 (-0.17, 0.34)	0.81
N+WSH vs N			0.05 (-0.07, 0.17)			0.25 (0.00, 0.51)	0.14

Supplementary Table 12: Effect modification with socio-economic status

	Low quintiles			Highest quintile			
Outcome	N	Mean (SD)	Mean Difference vs. Control (95% CI)	N	Mean (SD)	Mean Difference vs. Control (95% CI)	P for Interaction
**Communication z-score**							
Control	859	-0.09 (1.02)		240	0.31 (0.84)		
Water	457	0.03 (0.98)	0.11 (-0.01, 0.24)	108	0.34 (0.82)	0.09 (-0.14, 0.31)	0.82
Sanitation	431	0.17 (0.95)	0.25 (0.12, 0.39)	124	0.33 (0.93)	0.05 (-0.15, 0.25)	0.071
Handwashing	428	0.09 (0.98)	0.19 (0.06, 0.33)	115	0.58 (0.67)	0.27 (0.10, 0.44)	0.49
WSH	436	0.10 (0.95)	0.18 (0.05, 0.31)	111	0.33 (0.85)	0.04 (-0.17, 0.24)	0.24
Nutrition	432	0.13 (0.88)	0.22 (0.10, 0.34)	109	0.46 (0.71)	0.12 (-0.08, 0.31)	0.41
Nutrition+WSH	447	0.22 (0.90)	0.31 (0.20, 0.42)	106	0.40 (0.91)	0.12 (-0.11, 0.36)	0.15
N+WSH vs WSH			0.11 (-0.03, 0.25)			0.15 (-0.13, 0.43)	0.79
N+WSH vs N			0.10 (-0.04, 0.24)			-0.05 (-0.30, 0.21)	0.31
**Gross Motor z-score**							
Control	859	-0.07 (1.01)		240	0.27 (0.90)		
Water	452	-0.03 (0.94)	0.04 (-0.10, 0.17)	105	0.19 (0.88)	-0.05 (-0.29, 0.19)	0.51
Sanitation	429	0.07 (0.95)	0.15 (0.02, 0.28)	120	0.33 (0.82)	0.04 (-0.21, 0.29)	0.43
Handwashing	424	0.05 (0.95)	0.15 (0.02, 0.28)	111	0.30 (0.83)	0.00 (-0.20, 0.21)	0.21
WSH	433	0.11 (0.85)	0.17 (0.06, 0.28)	106	0.38 (0.90)	0.14 (-0.11, 0.38)	0.79
Nutrition	423	0.13 (0.92)	0.23 (0.12, 0.34)	105	0.38 (1.03)	0.05 (-0.19, 0.29)	0.15
Nutrition+WSH	440	0.13 (0.86)	0.20 (0.07, 0.33)	106	0.19 (1.21)	-0.06 (-0.32, 0.20)	0.081
N+WSH vs WSH			0.04 (-0.08, 0.16)			-0.21 (-0.52, 0.11)	0.15
N+WSH vs N			-0.03 (-0.19, 0.14)			-0.13 (-0.43, 0.18)	0.57
**Personal-social z-score**							
Control	859	-0.07 (1.01)		240	0.25 (0.90)		
Water	453	0.06 (0.92)	0.14 (0.01, 0.27)	104	0.33 (0.83)	0.13 (-0.11, 0.36)	0.90
Sanitation	421	0.24 (0.96)	0.31 (0.19, 0.43)	123	0.42 (1.02)	0.22 (-0.06, 0.49)	0.53
Handwashing	417	0.18 (0.93)	0.28 (0.15, 0.41)	111	0.57 (0.91)	0.31 (0.08, 0.53)	0.82
WSH	433	0.24 (1.01)	0.29 (0.17, 0.42)	105	0.43 (0.96)	0.22 (-0.05, 0.49)	0.65
Nutrition	422	0.18 (0.97)	0.25 (0.11, 0.38)	106	0.36 (0.95)	0.13 (-0.12, 0.38)	0.46
Nutrition+WSH	436	0.30 (0.94)	0.38 (0.27, 0.50)	102	0.49 (1.13)	0.26 (0.00, 0.52)	0.37
N+WSH vs WSH			0.07 (-0.07, 0.21)			0.07 (-0.25, 0.39)	0.97
N+WSH vs N			0.14 (-0.02, 0.30)			0.11 (-0.23, 0.45)	0.88
**Combined z-score**							
Control	859	-0.10 (1.02)		240	0.35 (0.83)		
Water	437	0.08 (0.87)	0.18 (0.07, 0.29)	102	0.38 (0.77)	0.09 (-0.14, 0.31)	0.43
Sanitation	414	0.26 (0.87)	0.36 (0.23, 0.49)	117	0.47 (0.80)	0.15 (-0.07, 0.36)	0.070
Handwashing	396	0.17 (0.89)	0.29 (0.17, 0.41)	106	0.66 (0.63)	0.29 (0.12, 0.45)	0.94
WSH	414	0.19 (0.92)	0.27 (0.15, 0.39)	105	0.48 (0.80)	0.18 (-0.06, 0.43)	0.53
Nutrition	401	0.20 (0.84)	0.31 (0.19, 0.43)	105	0.52 (0.77)	0.16 (-0.03, 0.36)	0.22
Nutrition+WSH	421	0.33 (0.78)	0.43 (0.32, 0.53)	101	0.49 (0.94)	0.17 (-0.06, 0.40)	0.040
N+WSH vs WSH			0.15 (0.01, 0.28)			0.00 (-0.30, 0.30)	0.36
N+WSH vs N			0.13 (0.00, 0.27)			-0.04 (-0.30, 0.22)	0.26

Supplementary Table 13: Distribution of target children developmental and maternal depressive scores by intervention groups, WASH Benefits site, Bangladesh

Outcome, Arm	Control	Water	Sanitation	Handwashing	WSH	Nutrition	WSHN
Maternal measures:							
**Maternal score for depressive symptoms**							
1-year-follow-up (Range 0 to 60)	11.54 (7.28)	11.65 (7.82)	11.44 (6.92)	11.18 (7.09)	11.42 (7.52)	10.13 (6.65)	10.92 (7.64)
2-year-follow-up(Range 0 to 60)	11.9 (7.28)	11.34 (6.96)	11.32 (7.04)	11.36 (7.09)	11.53 (7.05)	11.13 (7.02)	10.91 (7.13)
Child measures:							
**Stimulation received by child at home**							
1-year-follow-up (Range -7 to 33)	6.6 (3.03)	7.09 (3.17)	7.23 (3.02)	7.26 (3.18)	6.91 (2.93)	7.02 (3.05)	7.07 (3.08)
2-year-follow-up(Range -7 to 33)	10.07 (3.60)	10.88 (3.77)	10.39 (3.67)	10.55 (3.51)	10.12 (3.55)	11.07 (3.47)	11.17 (3.79)
**CDIs† scores understand**							
1-year-follow-up	15.23 (8.08)	15.95 (8.06)	15.91 (8.56)	16.1 (8.25)	16.08 (8.27)	16.14 (8.22)	16.23 (8.19)
2-year-follow-up	66.3 (11.43)	67.18 (12.7)	67.53 (11.71)	68.46 (11.18)	67.1 (12.89)	67.74 (11.86)	68.56 (12.01)
**CDIs† scores say**							
1-year-follow-up	5 (3.44)	5.12 (3.23)	5.35 (3.76)	5.41 (3.48)	5.19 (3.55)	5.24 (3.32)	5.28 (3.5)
2-year-follow-up	50.7 (20.97)	52.34 (21.3)	53.08 (20.76)	52.54 (20.94)	51.44 (21.93)	53.25 (20.63)	53.39 (21.91)
**EASQ‡ Communication score**							
2-year-follow-up	17.75 (5.8)	17.98 (5.69)	18.52 (5.67)	18.37 (5.73)	18.03 (5.71)	18.48 (5.23)	18.83 (5.52)
**EASQ‡ Gross motor skills score**							
2-year-follow-up	15.96 (3.14)	15.87 (3.1)	16.36 (2.95)	16.22 (3.17)	16.22 (3.46)	16.55 (3.11)	16.38 (3.08)
**EASQ‡ Personal-social skills score**							
2-year-follow-up	13.21 (3.31)	13.42 (3.36)	14.05 (3.55)	13.97 (3.56)	13.98 (3.82)	13.96 (3.59)	14.37 (3.54)
**EASQ‡ combined skills score**							
2-year-follow-up	46.92 (10.02)	47.27 (10.01)	48.93 (9.98)	48.56 (10.43)	48.23 (10.93)	48.98 (9.73)	49.58 (10.14)
**EF§- Tower test score**							
2-year-follow-up	6.27 (2.58)	6.47 (2.55)	6.25 (2.62)	6.51 (2.39)	6.34 (2.6)	6.39 (2.5)	6.53 (2.45)
**EF§- A not B score**							
2-year-follow-up	7.73 (2.45)	8.1 (2.16)	7.89 (2.3)	7.87 (2.28)	7.79 (2.41)	7.75 (2.36)	7.86 (2.29)

* All values are mean (SD) expect for maternal depressive symptoms score, where data are median (IRQ)

† The MacArthur-Bates Communicative Development Inventories

‡ EASQ= Extended Ages and Stages Questionnaire

§ EF=Executive function

Supplementary Table 14: ICC for different measures

Outcome	ICC
**Maternal depressive symptoms at year 1**	0.03
**HOME scale at year 1**	0.19
**Motor at year 1**	
Sitting alone	0.17
Hands and knees crawling	0.09
Standing with assistance	0.12
Walking with assistance	0.13
Standing alone	0.19
Walking alone	0.22
**CDI at year 1**	
Understand	0.13
Say	0.09
**EASQ at year 2**	
Communication	0.06
Gross Motor	0.07
Personal-social	0.05
Combined	0.06
**Maternal depressive symptoms at year 2**	0.05
**HOME scale at year 2**	0.16
**CDI at year 2**	
Understand	0.10
Say	0.08
**EF Tower test at year 2**	0.09
**EF A not B at year 2**	0.04

Supplementary Table 15: List of total Direct and Indirect measures in EASQ by domains

<table>
<tr><td colspan="2">Extended Ages and Stages Qquestionnaire-3
Observed three domains: Communication, Gross Motor &Personal social
Three age ranges are covered, each containing 12 questions per domains-
• <u>18-19 Months (17 months 16 days through 19 months 15 days)</u>
• <u>20-21 Months (19 months 16 days through 21 months 15 days)</u>
• <u>22-24 Months (21 months 16 days through 23 months 31 days)</u>
Items in each domain either maternal report or direct test/observation
Materials carried at home for direct tests included- Ball, Mirror, Small object (Car) & picture books</td></tr>
<tr><td rowspan="2">EASQ Communication</td><td>ASKED ITEMS (Communication)
• Does your child say two or three words that represent different ideas together, such as “See dog,” “Mommy come home,” or “Kitty gone”? (DO NOT COUNT WORD COMBINATIONS THAT EXPRESS ONE IDEA, SUCH AS “BYE-BYE,” “ALL GONE,” “ALL RIGHT,” AND “WHAT’S THAT?”) Please give an example of your child’s word combinations:
• Does your child say eight or more words in addition to “Mama” or “Dada”? (IF “YES,” ASK THE CAREGIVER TO NAME THE WORDS THE CHILD CAN SAY AND KEEP COUNT TO BE SURE THERE ARE AT LEAST EIGHT. DO NOT SUGGEST OR OFFER WORDS)
• Does your child say fifteen words or more in addition to “Mama” or “Dada”? (THE CHILD MUST SAY 15 WORDS OR MORE. IF THE CAREGIVER IS NOT SURE, SAY: “Let’s see if we can count the words together.” DO NOT GIVE EXAMPLES OF WORDS).
• Does your child correctly use at least two words like “me,” “I,” “mine,” and “you”? (THE PURPOSE OF THIS QUESTION IS TO KNOW WHETHER CHILD CAN IDENTIFY HIMSELF AND OTHERS WITH PRONOUNS.)
• Does your child imitate a two-word sentence? For example, when you say a two-word phrase, such as “Mama eat,” “Daddy play,” “Go home,” or “What’s this?” does your child say both words back to you? (MARK “YES” EVEN IF THE CAREGIVER RESPONDS THAT THE WORDS ARE DIFFICULT TO UNDERSTAND.)
• Does he point towards things he want?
• If you say something (2 words sentence) can he repeat after you? (Like-Go home, Ma goes, Baba plays, etc) (Some difficulties in understanding the words would do) (Probe- Does he repeat after you say something in front of him?) An example of the child’s sentence ---------
• Does your child make sentences that are three or four words long? For example, “Baby is sleeping,” “Where is the doggy [KITTY, CHICKEN, COW OR OTHER OBJECT]?” (SENTENCES DO NOT NEED TO BE GRAMMATICALLY CORRECT)
• Does your child say two or three words that represent different ideas together, such as “See dog,” “Mommy come home,” or “Kitty gone”? (DO NOT COUNT WORD COMBINATIONS THAT EXPRESS ONE IDEA, SUCH AS “BYE-BYE,” “ALL GONE,” “ALL RIGHT,” AND “WHAT’S THAT?”) Please give an example of your child’s word combinations:
• If asked to bring things he knows/uses from inside/outside the house, can he bring that? (Clothing, Ball, Glass etc that are not present at that room)
• Without giving him help by pointing or using gestures, if you ask your child to “Put the cup on the table” and “Put the bowl under the table,” does your child carry out both of these directions correctly? (THESE ACTIVITIES CAN BE MODIFIED TO REFLECT THE LOCAL CONTEXT. THE POINT IS THE CHILD UNDERSTANDS “ON” AND “UNDER.”)
• If asked, can he say his “Full Name”?</td></tr>
<tr><td>TESTED ITEMS (Communication)
Materials: Book
Direct Observations
• Does he point towards a picture in the book, scratch or try to take the picture?
• If you point to a picture of a ball (kitty, cup, hat, etc.) and ask your child, “What is this?” does your child correctly name at least one picture? (HE NEEDS TO NAME ONLY ONE PICTURE CORRECTLY.)
• Without giving him clues by pointing or using gestures, can your child carry out at least three of these</td></tr>
</table>

	kinds of directions? (PLEASE ASK ABOUT THESE DIRECTIONS ONLY. OBJECTS IN BRACKETS CAN BE SUBSTITUTED WITH THOSE MORE FAMILIAR TO THE CHILD.)<br>a. “Put the [TOY] on the table.” b. “Close the door.” c. “Bring me a [TOWEL].”<br>d. “Find your [COAT].” e. “Take my hand.” f. “Get your [BOOK].”<br>• When you ask her to point to her nose, eyes, hair, feet, ears, and so forth, does your child correctly point to at least seven body parts? She can point to part of herself, you, or a doll.<br>• If not shown but asked, can he correctly show the picture of something from the book? (Like-Where is the cat, show the dog, etc. At least one will do)<br>• When looking at a picture book, does your child tell you what is happening or what action is taking place in the picture? For example, if you ask, “What is the dog (or boy) doing?” does he respond, “Barking,” “Running,” “Eating,” or “Crying”?
EASQ Gross Motor Skills	**ASKED ITEMS (Gross Motor Skills)**<br>• Does your child walk well and seldom fall?<br>• Does your child climb on an object such as a chair (OR ROCK, ETC.) to reach something she wants?<br>• Does your child walk down stairs (AT LEAST ONE STEP) if you hold onto one of her hands?<br>• Does your child run fairly well, stopping herself without bumping into things or falling<br>• Does your child walk either up or down at least two steps by himself? (CHECK “YES” EVEN IF HE HOLDS ONTO THE WALL OR RAILING.<br>• Can he, without holding anything, fold his knees, pick up anything from the floor and stand up again (By himself)?<br>• Can he climb up steps? (Taking support of wall or railing, right foot on one step, then left on the other-in this way)<br>• Does your child stand on one foot for about 1 second without holding onto anything?<br>• Can he climb up any furniture or anything by himself? I.e. Bed, Chair, etc.<br>• Does your child walk up stairs, using only one foot on each stair? That is, the left foot is on one step, and the right foot is on the next. (HE MAY HOLD ONTO THE RAILING OR WALL.)
	**TESTED ITEMS (Gross Motor Skills)**<br>**Materials: Ball**<br>**Direct Observations**<br>• When you show him how to kick a large ball [OR OTHER OBJECT], does your child try to kick the ball by moving his leg forward or by walking into it? (IF CHILD ALREADY KICKS A BALL OR OTHER OBJECT, CHECK “YES” FOR THIS ITEM.)<br>• Without holding onto anything for support, does your child kick a ball [OR SOME OTHER OBJECT] by swinging his leg forward?<br>• Does your child jump with both feet leaving the floor at the same time?<br>• Does your child jump forward at least 3 inches with both feet leaving the ground at the same time?<br>• With both foot, can he jump forward at least 6 inches (Show the measurement with hand)?<br>• Without holding anything, can he swing his feet from back to front and kick the ball?<br>• While standing, does your child throw a ball overhand by raising his arm to shoulder height and throwing the ball forward? (DROPPING THE BALL, LETTING THE BALL GO, OR THROWING THE BALL UNDERHAND SHOULD BE SCORED AS “NOT YET.”)
EASQ Personal-Social	**ASKED ITEMS (Personal-Social Skills)**<br>• Does your child come to you when he needs your help with something, such as [WINDING UP A TOY] or opening something [A BOTTLE, BANANA, ORANGE, SOME OTHER KIND OF FOOD]?<br>• Does your child copy the activities you do, such as [WASH DISHES, COOK, PREPARE FOOD, CARE FOR ANIMALS, ETC. OR…] wipe up a spill, sweep, shave, or comb hair? [NOTE: CHILDREN DO NOT HAVE TO DO THE ACTIVITIES PERFECTLY. THE POINT IS THEY TRY TO COPY THEM.]<br>• Does your child drink without help from a cup or glass, putting it down again with little spilling? [CHILD CAN DRINK FROM A CUP WITHOUT SPILLING TOO MUCH]<br>• Is he able to feed himself with a spoon or by his hands (without spilling)? (If the answer is yes, ask what does he eat?)<br>• Is he able to feed himself with a spoon (some spilling acceptable)? (THE POINT IS THE CHILD CAN FEED SELF WITH LITTLE SPILLING)<br>• Is he able to feed himself with a spoon or by his hands (without spilling)? (If the answer is yes, ask what does he eat?)

- Does he hug human or animal like stuff toys?
- Does he try to do the same when you take off his shoes, socks, caps?
- To grab your attention or to show you something, does he pull your hand or clothing?
- When playing with either a stuffed animal or doll [OR ITEM REPRESENTING THIS], does your child pretend to rock it, feed it, change its diapers, put it to bed, and so forth? (THE PURPOSE OF THIS QUESTION IS WHETHER CHILD CARES FOR THE DOLL OR STUFFED ANIMAL AS IF IT WERE A PERSON. BOYS MAY NOT BE ENCOURAGED TO DO THIS. WE RECOMMEND KEEPING THE ITEM AND DROPPING IT LATER, DURING ANALYSES, IF IT APPEARS TO NOT WORK WITH BOYS AND GIRLS ALIKE.)
- Does your child call herself “I” or “me” more often than her own name? For example, “I do it,” more often than “[CHILD] do it.”
- Does your child put on a coat, jacket, or shirt by himself?
- When you to put a loose pant or pajama on him, does he pull it up himself from his feet to waist?

TESTED ITEMS (Personal-Social Skills)
Materials: Mirror & small cart
Direct Observations

- While looking at himself in the mirror, does your child offer a toy to his own image?
- When he is looking in a mirror and you ask, “Who is in the mirror?” does your child say either “Me” or his own name?
- If you do any of the following gestures, does your child copy at least one of them? (GESTURES MUST BE THESE EXACTLY; DO NOT SUBSTITUTE OTHER GESTURES.)
  a. Open and close your mouth.
  b. Blink your eyes.
  c. Pull on your earlobe.
  d. Pat your cheek
- Does your child push a little shopping cart, stroller, or wagon, [OR OTHER OBJECT WITH WHEELS] steering it around objects and backing out of corners if he cannot turn?

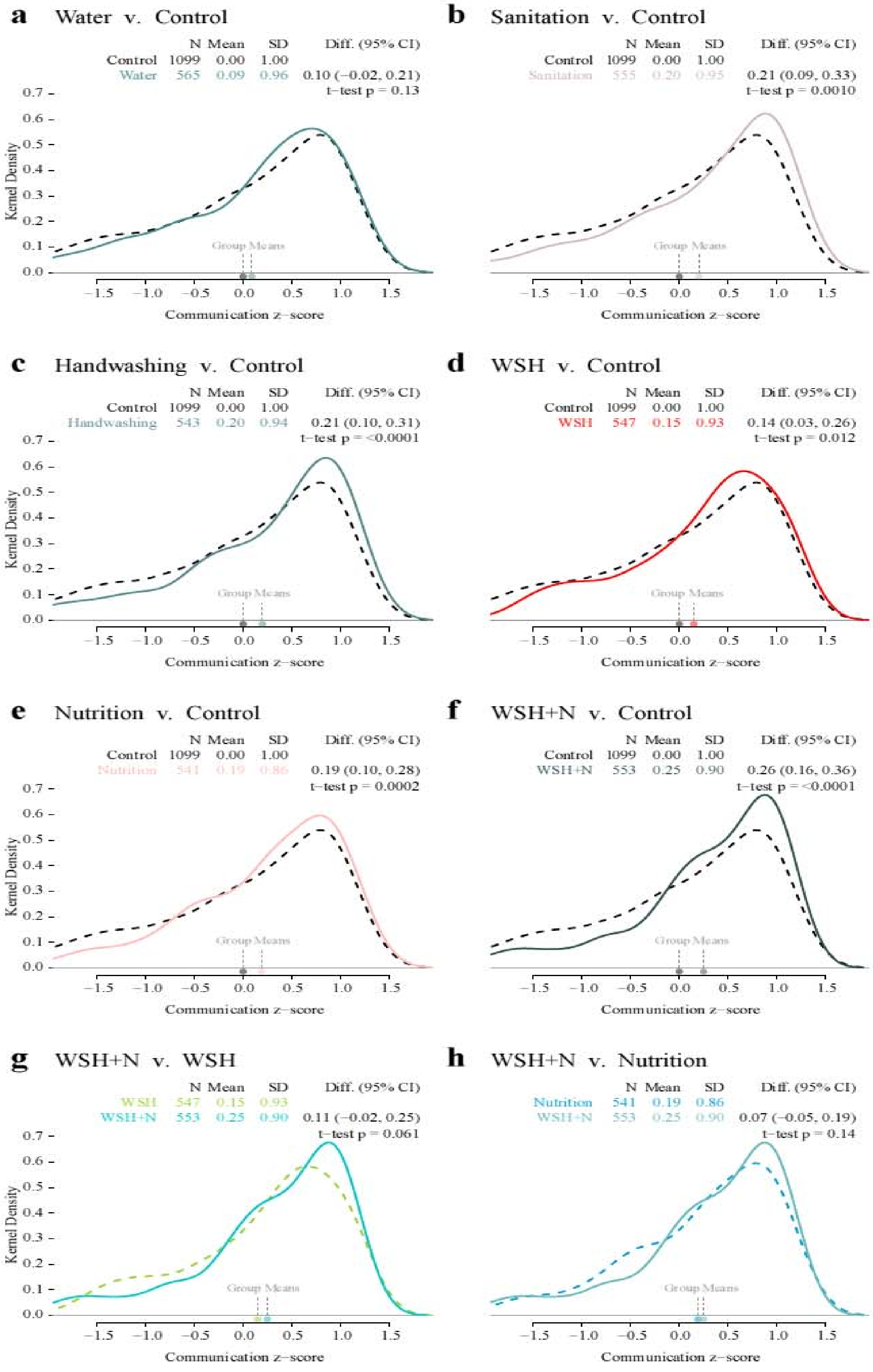

Supplemetary figure 1: Caption on the next page.

Intervention effects on communication Z-scores after 2 years of intervention. Kernel density plots summarize the distribution of communication Z-scores of index children who were born into the study and were between 21-30 months (median=26, IRQ= 24, 27) at the time of measurement. In each panel, a dashed line illustrates the comparison group distribution and a solid line illustrates the active comparator distribution. **a-f**, each active intervention arm compared against the double sized control arm; **g-h**, combined water, sanitation, handwashing (WSH) plus nutrition compared against either nutrition alone or WSH alone. Mean differences and CIs are from an unadjusted analysis using generalized linear models that considered pair matching and block level clustering. T-test p-values test whether differences in group means are different from zero using a paired t-test and cluster level means.

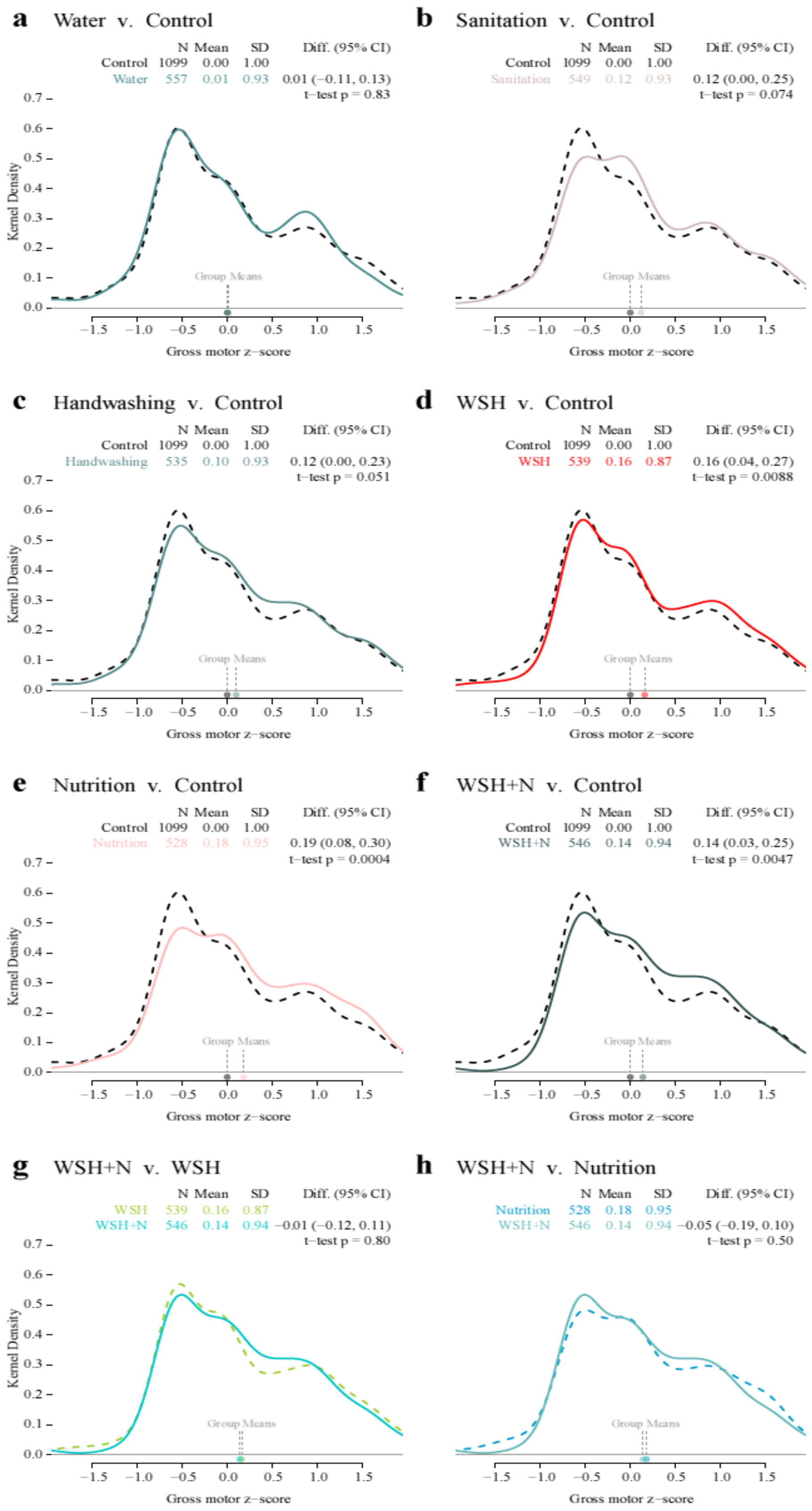

Supplementary figure 2: Caption on the next page.

Intervention effects on gross motor Z-scores after 2 years of intervention. Kernel density plots summarize the distribution of gross motor Z-scores of index children who were born into the study and were between 21-30 months (median=26, IRQ= 24, 27) at the time of measurement. In each panel, a dashed line illustrates the comparison group distribution and a solid line illustrates the active comparator distribution. **a-f**, each active intervention arm compared against the double sized control arm; **g-h**, combined water, sanitation, handwashing (WSH) plus nutrition compared against either nutrition alone or WSH alone. Mean differences and CIs are from an unadjusted analysis using generalized linear models that considered pair matching and block level clustering. T-test p-values test whether differences in group means are different from zero using a paired t-test and cluster level means.

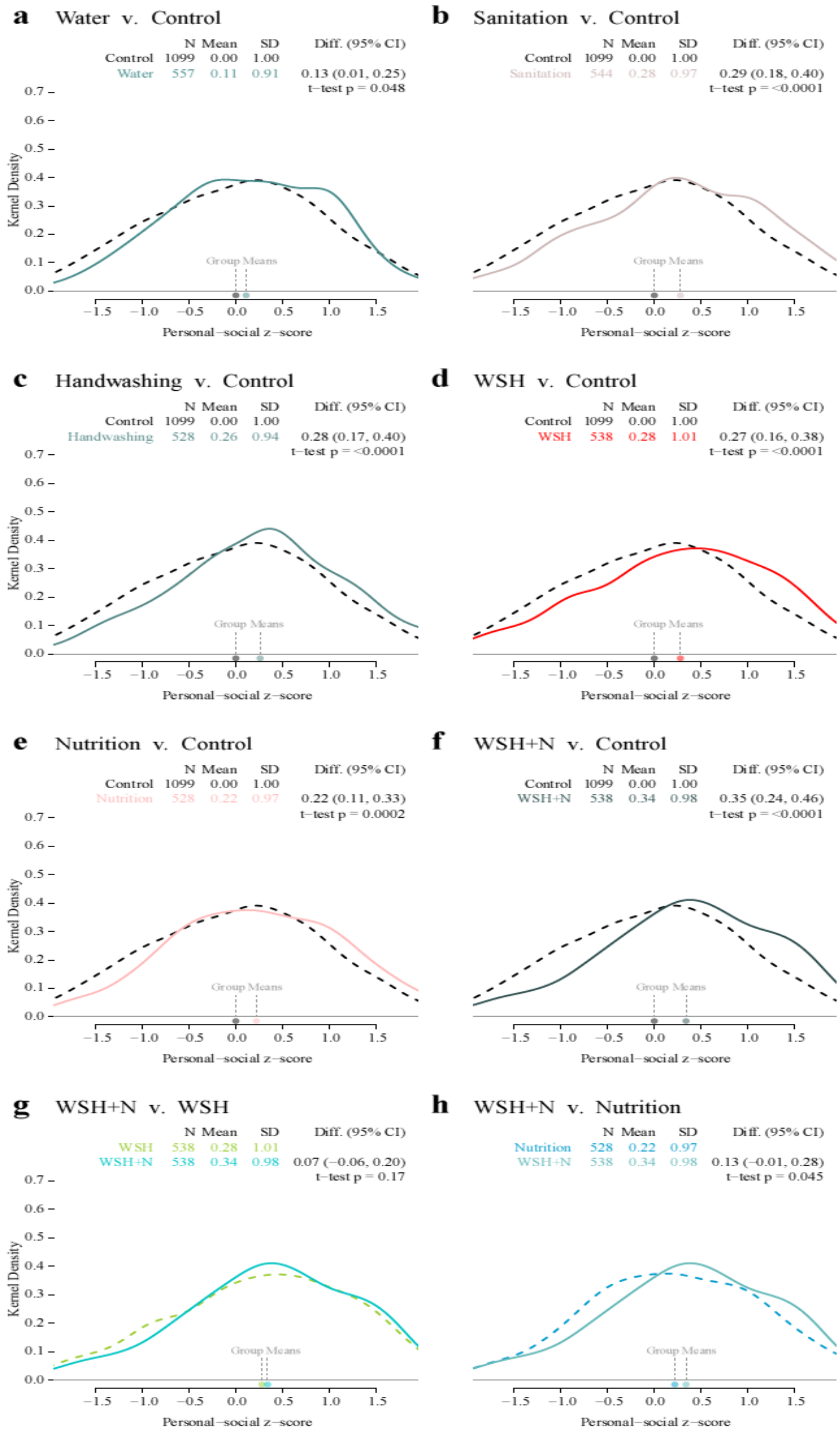

Supplemetary figure 3: Caption on the next page.

Intervention effects on personal-social Z-scores after 2 years of intervention. Kernel density plots summarize the distribution of personal-social Z-scores of index children who were born into the study and were between 21-30 months (median=26, IRQ= 24, 27) at the time of measurement. In each panel, a dashed line illustrates the comparison group distribution and a solid line illustrates the active comparator distribution. **a-f**, each active intervention arm compared against the double sized control arm; **g-h**, combined water, sanitation, handwashing (WSH) plus nutrition compared against either nutrition alone or WSH alone. Mean differences and CIs are from an unadjusted analysis using generalized linear models that considered pair matching and block level clustering. T-test p-values test whether differences in group means are different from zero using a paired t-test and cluster level means.
